# Mass-Rearing of *Drosophila suzukii* for Sterile Insect Technique Application: Evaluation of Two Oviposition Systems

**DOI:** 10.3390/insects10120448

**Published:** 2019-12-12

**Authors:** Fabiana Sassù, Katerina Nikolouli, Silvana Caravantes, Gustavo Taret, Rui Pereira, Marc J. B. Vreysen, Christian Stauffer, Carlos Cáceres

**Affiliations:** 1 BOKU, Department of Forest and Soil Sciences, University of Natural Resources and Life Sciences, 1190 Vienna, Austria or K.Nikolouli@iaea.org (K.N.); christian.stauffer@boku.ac.at (C.S.); 2 Insect Pest Control Section, Joint FAO/IAEA Division of Nuclear Techniques in Food and Agriculture, Wagramerstrasse 5, P.O. Box 100, A-1400 Vienna, Austria; silvana.caravantes@gmail.com (S.C.); R.Cardoso-Pereira@iaea.org (R.P.); M.Vreysen@iaea.org (M.J.B.V.); C.E.Caceres-Barrios@iaea.org (C.C.); 3 Instituto de Sanidad y Calidad Agropecuaria ISCAMEN, 3050 Mendoza, Argentina; gustavotaret@yahoo.com.ar

**Keywords:** wax panel system, netted system, spotted wing drosophila, pest management, female oviposition behavior

## Abstract

*Drosophila suzukii* (Diptera: Drosophilidae) is an invasive pest of a wide range of commercial soft-skinned fruits. To date, most management tactics are based on spraying of conventional and/or organic insecticides, baited traps, and netting exclusion. Interest has been expressed in using the sterile insect technique (SIT) as part of area-wide integrated pest management (AW-IPM) programs to control *D. suzukii* infestations. Mass-rearing protocols are one of the prerequisites for successful implementation of the SIT. To establish mass-rearing methods for this species, two different egg-collection systems were developed and compared with respect to the number of eggs produced, egg viability, pupa and adult recovery, adult emergence rate, and flight ability. Female flies kept in cages equipped with a wax panel produced significantly more eggs with higher viability and adult emergence rate, as compared to the netted oviposition system. The wax panel system was also more practical and less laborious regarding the collection of eggs. Furthermore, the wax panel oviposition system can be adapted to any size or design of an adult cage. In conclusion, this system bears great promise as an effective system for the mass production of *D. suzukii* for SIT.

## 1. Introduction

The successful implementation of the sterile insect technique (SIT) as part of an area-wide integrated pest management (AW-IPM) approach against a target pest species requires the development of technologies such as the mass-rearing of the target species, the optimal irradiation doses to sterilize the males and the females, adequate quality control protocols, and packing, shipping, holding, and release procedures [1,2,3,4]. Efficient mass-rearing methods are required to allow for continuous and stable production of sufficient numbers of insects at an economically viable cost [5].

Adapting wild insects to artificial laboratory rearing conditions is the first step in development of a mass-rearing system. The adaptation process should avoid adverse effects like modification of the natural behavior of the insect, resulting in poor field performance; inadequate sexual competitiveness; or incompatibility with the wild target insect population [6]. Mass-rearing of fruit flies comprises several steps, starting with the adaptation of the females’ oviposition behavior under artificial conditions to maximize production and ensure optimal quality of the eggs [7,8]. Female oviposition behavior plays an essential role in the development of suitable oviposition devices to achieve the required production goal [9,10]. When a large number of an insect species is reared, natural hosts are no longer practical as an egg-collection system. Consequently, a functional and cost-efficient method must be developed for females to oviposit in artificial substrates or devices, allowing the best possible egg recovery [11]. Under artificial conditions, the adaptation of a female’s oviposition behavior may occur after several generations due to the low heritability or genetic complexity involved in oviposition behavioral traits [12,13]. The adaptation of the female’s oviposition from the natural host to an artificial diet and from an artificial diet to a non-diet device results in bottleneck events that may reduce the colony genetic variability in addition to selecting for a specific rearing behavior [14].

To date, 13 species of Tephritidae are mass-reared worldwide for pest-control purposes [15], and a practical artificial oviposition method has been developed based on the biology and ethology of each species [16].

Artificial egg-collection systems for large-scale rearing of insects are highly variable, e.g., parasitoid species lay eggs on the natural host egg, larva, or pupae [17,18] and some species of mass-reared mosquitoes and Lepidoptera lay eggs on moist or dry paper substrates where eggs are either manually removed by a brush [19,20,21] or placed directly onto the larval diet [22]. Perforated bottle or domes simulating fruits are used as an oviposition device for some species of fruit flies (*Ragoletis cerasi*, *Bactrocera* spp., *Dacus* sp.) [23,24,25], whereas a broad range of either flat membranes, panels, or nets with different composition, size, color, and thickness are used as a collection method (“egg-dropping”) for the Mediterranean fruit fly *Ceratitis capitata* and *Anastrepha* spp. [26,27].

The “egg-dropping” technique is the most common method applied in fruit fly mass-rearing facilities worldwide [9,28]. More than two billion eggs of *C. capitata* are collected weekly with this system (Cáceres C., personal communication). Females oviposit eggs through the netting and the eggs are collected in a metallic or PVC container filled with water. The *C. capitata* colony is a strain that has been adapted to laboratory conditions for decades, and females can therefore lay eggs through this artificial system in the same quantity as the natural host without any external stimulants. Therefore, the amount of eggs produced and collected can be easily quantified, which is important for the estimation of colony production and management.

Spotted wing drosophila (SWD) *Drosophila suzukii* (Matsumura) (Diptera: Drosophilidae) is a destructive insect pest of fruit crops. Female *D. suzukii* possess a saw-shaped ovipositor that allows them to pierce of the skin of ripening fruit, contrary to other Drosophilidae flies that prefer laying eggs in decaying fruits [29,30,31]. *D. suzukii* females oviposit eggs and larvae develop on a large number of different soft skinned hosts [32,33], making many fruit crops in Europe and America susceptible to infestation with high economic losses [34,35,36]. These losses are mitigated by applying insecticides [37], performing field sanitation [38], mass-trapping [39,40], and introducing mechanical impediments such as nets to physically protect the fruit [41,42].

As a result of the invasion of *D. suzukii* into the European and American continents, government agencies and growers have been looking for alternative control methods that are more efficient than the existing methods and more benign to the environment [36,43]. Therefore, different research groups have been focusing on the development of an SIT package for the control of *D. suzukii*, making use of the experience and knowledge that has been accumulated over the years with Tephritidae species.

The main objective of this study was to develop an efficient, practical, and economically viable oviposition system that would allow production of eggs under artificial conditions for large-scale *D. suzukii* production for SIT application. In this study, a comparison was made of two oviposition systems with respect to the total egg production, egg viability, egg to pupa developmental time, pupa and adult recovery, and the quality of the produced adults as assessed by adult emergence and flight ability.

## 2. Materials and Methods

### 2.1. Drosophila suzukii Colony

The *Drosophila suzukii* used in this study came from a colony that was established from flies collected in San Michele All’Adige, Trento (Italy) in 2014, and reared at the Insect Pest Control Laboratory (IPCL) in Seibersdorf, Austria. Adult flies were maintained in aluminum-framed cubic cages (45 × 45 × 45 cm) that were loaded with approximately 30,000 pupae per cage. Pupae volume was measured in a graduated cylinder (1 mL = 220 pupae). Emerging adult flies had unlimited access to water and a diet consisting of a blend of sugar and enzymatic hydrolyzed yeast at a 3:1 ratio. Subsequently, insects were transferred to larval diet consisting of 25.7% wheat bran, 6.4% brewer yeast, 11.9% sugar, 0.4% sodium benzoate, 0.4% nipagin, and 55.1% water. They were kept at a temperature of 22 ± 5 °C, 65 ± 5% RH, and a 14:10 h L:D photoperiod.

### 2.2. Oviposition Systems

**Figure 1:**
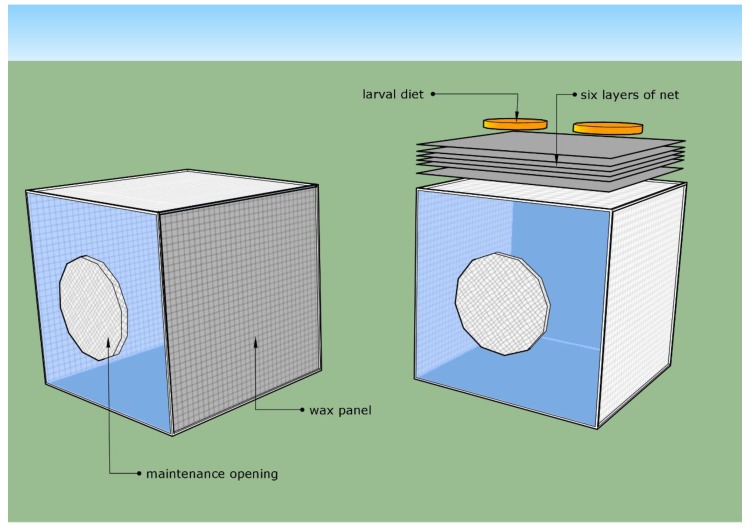
Design of rearing cages. (**Left**) Wax oviposition system; (**right**) netted oviposition system.

The “netted oviposition system” was developed and has been used as a rearing method for *D. suzukii* at the IPCL since 2017. Thereafter, “the wax panel system” was developed as a more cost-effective oviposition system (Figure 1). Two colonies were maintained with both systems for ten generations, and after this period of adaptation, a comparison was made in terms of production efficiency and quality.

#### 2.2.1. Net System

The net system consisted of a cubic aluminum frame and three transparent PVC plates, i.e., one each for the floor, the back, and the front. The front panel had a round hole (200 mm diameter) for introducing and removing water, adult diet, and pupae. Two sides of the cage were covered with mesh netting (mesh hole size: 0.22 × 0.22 mm) to allow for air exchange. The top of the cage consisted of one fixed internal mesh net (hole size: 1.0 × 1.5 mm), and six removable layers of synthetic black nylon net (Art. Velo, Sirio Tendaggi S.r.l., Magnago, Italy) (hole size: 0.22 × 0.22 mm) were placed on the top of the cage and used as an oviposition substrate. Petri dishes filled with the larval diet were used as a stimulant and placed on the top of the sixth layer of the synthetic black nylon, allowing females to lay eggs through the first five layers without having direct contact with the diet. This system allowed the collection of eggs that were trapped among the first five layers of net, avoiding contact with the artificial larval diet which only served as an oviposition attractant. Every day, the six netted layers were removed from the top of the cage and the eggs were collected by washing the first five layers with tap water. The sixth, top layer was changed daily. After the daily egg collection, the quantity of eggs produced was assessed volumetrically using graduated cylinders.

#### 2.2.2. Wax Panel

The wax panel system was similar to the design of the netted system. However, in this system, one of the lateral mesh facets of the cage was used as an oviposition site. This layer comprised an internal net with a mesh hole size of 1.0 × 1.5 mm, and an external layer of a finer black net with a mesh hole size of 0.22 × 0.22 mm. Both nets were fully immersed in a hot liquid solution containing 3% beeswax, 20% liquid paraffin, 49% solid paraffin (52–54), and 28% glycerin, and left at room temperature (22 ± 5 °C) to solidify. As a result, the two layers of nets were stuck together to form a single sealed waterproof layer. The internal net with the larger mesh hole size served as a resting and support area, while the external one served as an oviposition surface. The waxed panel was dabbed with guava juice daily to stimulate female oviposition. Females oviposited by inserting the ovipositor directly through the waxed panel. Due to the filament of *D. suzukii* eggs, the eggs stuck to the panel. The oviposited eggs were washed off the wax panel daily with water and collected in the containers placed along the bottom of cage.

### 2.3. Evaluation of the Artificial Oviposition Systems

To assess the two oviposition systems, 33 mL of pupae (approximately 7000 pupae) were placed in each cage (30 × 30 × 40 cm) on moist filter paper to prevent desiccation and to facilitate emergence. Emerged adults had unlimited access to water and a blend of sugar and yeast hydrolysate enzymatic (MP Biomedicals, Eschwege, Germany) (3:1) as adult diet [44]. All experiments were carried out under the same environmental conditions as the rearing colony. The same number of pupae was used for each experiment and they were replicated five times. Replicates were implemented at different times and thus five consecutive generations were evaluated.

### 2.4. Egg Production and Larvae Rearing

In both oviposition systems, eggs were filtered after collection, and the total volume of eggs (in mL) was recorded daily. Eggs were then incubated on moist black filter paper and maintained in Petri dishes. After 24 h, eggs were washed with water from the Petri dishes and placed on the artificial larval diet.

Additionally, samples of 100 eggs from both the netted and wax panel systems were sampled daily, incubated on a moist black cloth, and placed in Petri dishes filled with the rearing diet. The following parameters were assessed:

- Egg hatch: closed eggs were counted 48 hours after the collection day;
- Egg to pupa time: 4–5 days after egg collection, Petri dishes were checked for pupae (cryptocephalic pupae) to assess the duration of larval development;
- Pupa recovery: about 11 ± 1 days after the egg-collection day, pupae were removed from the diet, counted, placed on moist paper, and left in sealed boxes without water and food for adult emergence;
- Adult production: once dead, the emerged adults were removed from the boxes and then counted and sexed under stereoscope.

### 2.5. Adult Emergence and Flight Ability

The total egg production for each cage (with the exception of the samples mentioned above) was separately transferred daily to the larval diet. To allow larval development, about 0.3 mL of eggs was transferred to 80 ± 5 g of larval diet. Approximately 11 ± 1 days after the egg transfer, pupae had completed their development and were removed from the diet. These were counted and a sample was taken for evaluation of the quality control parameters.

For each oviposition system, 10 pupae were sampled daily (860 pupae in total for each oviposition system) and placed in a Petri dish to assess adult emergence. The experiment was replicated five times, and each replicate represented a different generation. The percentage of adult emergence was recorded to estimate the number of adults available in the next generation.

For each oviposition system, 10 pupae were sampled daily (860 pupae in total for each oviposition system) to assess the ability of adults to fly (hereafter: “flight ability” or “flyers”). The experiment was replicated five times, with each replicate representing a different generation. Flight ability tests were carried out to assess the quality of emerged adults. A black PVC cylinder (10 cm height and 8.4 cm internal diameter) was used for the test, as described in the FAO/IAEA/USDA quality control manual [2]. Pupae were placed inside a paper ring in a Petri dish and covered with a black PVC cylinder coated internally with talcum powder to prevent emerged adults crawling out of the cylinder. Flies outside the tubes were removed every hour to avoid re-entry into the cylinders. No food or water was provided during the experiments.

Adult emergence and flyers were calculated as described in the FAO/IAEA/USDA quality control manual [2].

### 2.6. Data Analysis

The data were analyzed in R, version 3.4.4 [45] using the package lme4 for all models [46]. Linear regression followed by Kruskal–Wallis test was used to test the effect of the generations (replicates) on the egg production for each egg-collection system. Statistical tests on the egg production and egg to pupa time were performed using the Mann–Whitney–Wilcoxon test (hereafter: “W”). The egg hatch was analyzed using a linear mixed model where the two egg-collection systems was included as a fixed effect and the replicates were included as a random effect. The proportions of pupa recovery and adult production were determined as the number of pupae and adults, respectively, per total number of egg hatch. The pupae recovery and adult production proportions were analyzed using a linear mixed model where the two egg-collection systems and the day of collection (number of days since the given replica started) were included as fixed effects and the replicates were included as random effect. To analyze the quality control parameters (adult emergence and flight ability), we used a linear mixed model where the two egg-collection systems were included as a fixed effect and the replicates were included as a random effect. In all data, the mean ± standard error is reported. The statistical analyses (Supplementary Materials, S1) and raw data (Supplementary Materials, S2, S3), are available online.

## 3. Results

### 3.1. Production Parameters

Initially, the number of eggs per volume unit was assessed to enable volumetric measurements of the eggs. On average, 2303.8 ± 269.6 eggs were counted in a volume of 0.1 mL over seven replicates.

**Figure 2:**
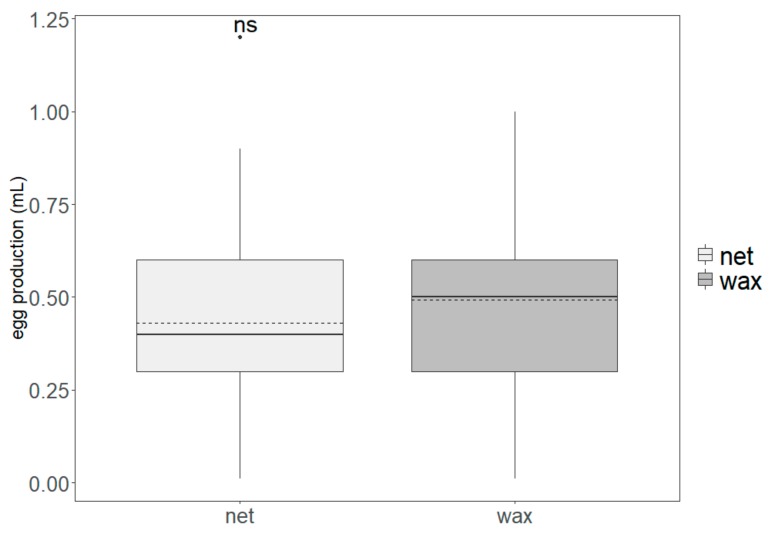
Volume of eggs of *Drosophila suzukii* produced in cages with netted and wax panel oviposition systems. Boxes indicate the interquartile range, bold lines indicate medians, dashed lines indicate means, whiskers indicate minimum and maximum values, and the dot indicates an outlier. Non-significant differences between treatment groups are indicated with “ns” (*p* > 0.05).

The replicates (generations) had no effect on the egg production for either egg-collection system (Kruskal–Wallis: *χ*^2^ = 6.0276, *df* = 9, *p* = 0.7372). The daily egg production was not significantly different for the two oviposition systems (*W*: 3739.5, *p* = 0.0521, N = 182), with an average of 0.48 ± 0.24 mL of eggs collected daily with the wax oviposition system and 0.43 ± 0.21 mL with the netted system (Figure 2).

**Figure 3:**
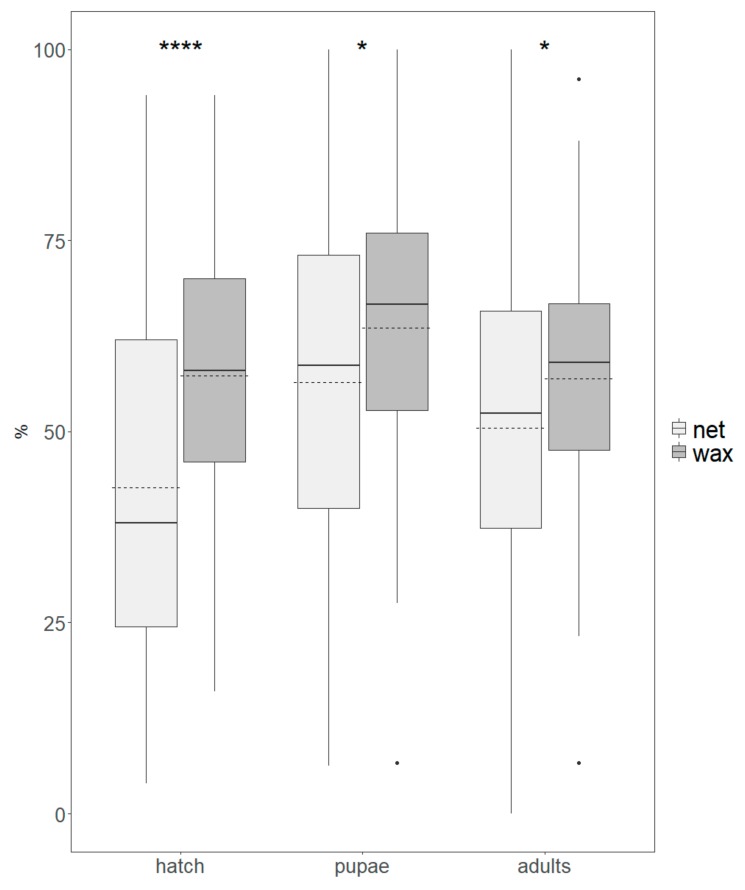
Percentages of egg hatch, pupa recovery, and adult production of *Drosophila suzukii* from netted (light grey) and wax (dark grey) cages. Boxes indicate the interquartile range, bold lines indicate medians, dashed lines indicate means, whiskers indicate minimum and maximum values, and dots indicate outliers. Significant differences between treatment groups are indicated with asterisks (* *p*< 0.05; **** *p* < 0.0001).

The egg hatch was significantly higher for the wax system compared to the netted system (*t-value* = 5.30, *df* = 182, *p* < 0.0001). The average of the daily percentage of egg hatch (± S.E.) was 42.7 ± 22.6 and 57.3 ± 16.9 for the net and wax cages, respectively (Figure 3).

For both egg-collection systems, the time from egg to pupation was similar (*W* = 2883.5, *p* = 0.1538, N = 161) with an average of 7.4 ± 0.8 and 7.6 ± 0.8 days from the egg-collection day to the first pupa for the net and wax system, respectively.

Pupae recovery and adult production proportions were significantly higher for the wax panel system compared to the netted system, i.e., a mean of 63.5 ± 17.1 and 56.5 ± 21.7 of pupae for the wax panel and netted system, respectively (*t-value* = 2.583, *df* = 182, *p* = 0.0106) and 56.9 ± 16.3 adults for the wax panel, compared with 50.4 ± 21.6 adults for the netted system (*t-value* = 2.435, *df* = 182, *p* = 0.0158) (Figure 3).

### 3.2. Quality Assessment

The average percentage of adults emerged from pupae produced in the netted oviposition system was 74.9% ± 21.2%, compared with an average of 85.5% ± 12.5% adults emerged in the wax panel system. The average number of adults that were able to fly was higher in the wax system compared with the netted system (77.6% ± 16.5% and 66.5% ± 24.1%, respectively).

**Figure 4:**
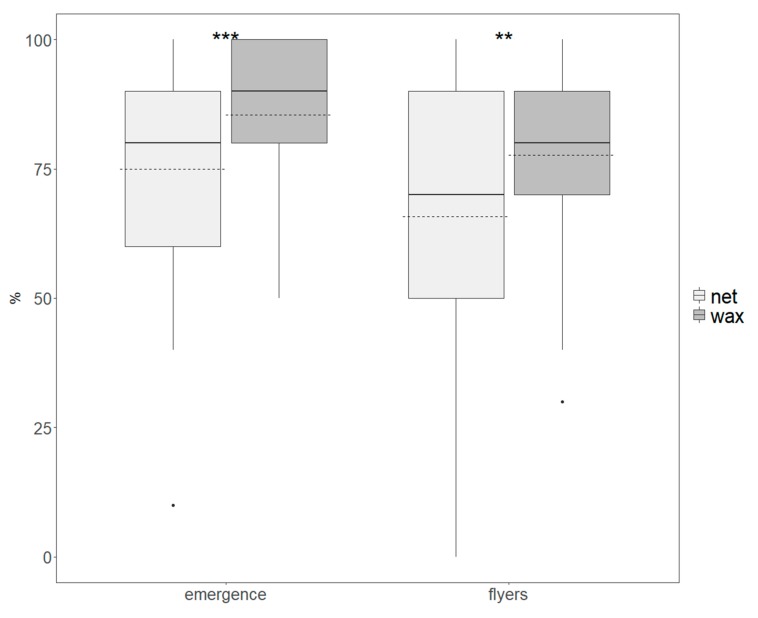
Adult emergence (**left**) and flyers (**right**) of *Drosophila suzukii* produced by the netted (light grey) and wax panel (dark grey) oviposition systems. Boxes indicate the interquartile range, bold lines indicate medians, dashed lines indicate means, whiskers indicate minimum and maximum values, and dots indicate outliers. Significant differences between treatment groups are indicated with asterisks (** *p* < 0.001; *** *p* < 0.0001).

Both the adult emergence and flyer data showed that there were significant differences between the netted and wax system (*t-value* = 4.101, *df* = 163, *p* = 0.0001, and *t-value* = 3.700, *df* = 163, *p* = 0.0003, respectively) (Figure 4).

## 4. Discussion

An efficient egg-collection system is a prerequisite for the development of a cost-effective and practical mass-rearing system for insects. In tephritids that oviposit eggs in fresh fruits, several methods have been assessed and developed for artificial mass production. Those methods range from perforated plastic egg bottles that contain fruit juice as attractants for *Batrocera* species to mesh plastic nets for *Ceratitis* species and silicon panels for *Anastrepha* species [25,47]. All these systems enable the designing of a rearing cage prototype that enables easy handling and cleaning [48,49].

The SIT can be integrated into the IPM practices for the control of *D. suzukii*, but for the mass-rearing process, it is necessary to develop a cost-effective artificial egg-collection system. We have developed two potential systems that may be used as routine protocol for the artificial egg production of *D. suzukii*. In this study, we compared netted and wax panel systems. The rearing in both systems was artificial and eggs were collected without the need for further separation from any natural or semi-natural host substrate. Consequently, all eggs oviposited were quantified, allowing an accurate estimate of total colony production.

The wax panel system and as the netted system were based on the same principle as the widely used “egg-dropping” technique [10]. This method has proven to be a practical and productive oviposition system for the mass-rearing of various tephritid species [25,27], having been routinely used for more than 30 years in mass-rearing facilities [50].

The wax panel system was more efficient than the netted system with respect to egg hatch and number of pupae and adults produced. Previous studies have shown that the number of eggs oviposited by females can be influenced by the artificial rearing method, reducing the quantity and quality of all development stages [51]. The results revealed that the netted method caused damage to a relatively high percentage of eggs during oviposition or during the egg-collection process. The risk of egg damage is important in *D. suzukii*, of which the eggs have two respiratory appendages that can easily be harmed.

In this study, the quality of the flies produced with the two methods was assessed. In tephritid fruit flies as well as in other insect species, mass-rearing conditions often compromise the quality of adults, e.g., damaged wings or shortened lifespan, reducing the performance of males after release in the field [52]. Our results showed that flies produced using the wax panel system were of better quality with regards to adult emergence and flight ability compared with the netted system. Therefore, production efficiency and quality of the *D. suzukii* was higher in the wax panel system.

Following an initial stressful period due to host restriction or deprivation, egg-laying behavior may be altered, resulting in females that can oviposit in unusual oviposition sites. Hence, after several generations and due to selection pressure, females can adapt their egg-laying pattern to artificial rearing conditions [53]. Therefore, considering the relatively short adaptation time of the colony to the two rearing systems, there is potential for future improvement of the *D. suzukii* mass-rearing system. Large quantities of high-quality sterile males are crucial to ensure optimal implementation of the SIT as a pest control tactic for *D. suzukii*. In spite of the fact that specific data were not collected in this study, our experience suggests that the use of a wax panel as an artificial oviposition system could facilitate the daily practices of colony maintenance and thus become more cost-effective for the mass-rearing of *D. suzukii*.

This study presents an important improvement in the development of the mass-rearing cage for *D. suzukii* regarding oviposition. An ultimate rearing cage design to optimize egg female production would require additional investigations on optimal cage size, adult density, quality, and quantity of adult food. It will be necessary to undertake more research and to assess the impact of different larval diets, establishing a pupae separation system, and refining the environmental parameters required for maintaining the colony.

## 5. Conclusions

This was the first study to develop and compare two oviposition systems for the rearing of *D. suzukii*. It was also the first to evaluate an oviposition system in large-scale rearing cages for this pest. The two oviposition systems were tested and the wax panel was more efficient as well as more practical compared with the netted system. Further studies will be crucial for the development of the whole SIT technology package targeted at *D. suzukii*.

## Supplementary Materials

The following are available online at https://www.mdpi.com/2075-4450/10/12/448/s1, S1: Statistical analyses, S2: Dataset all parameters, S3: Dataset quality control parameters.
