# Supplementary material for: Mass-Rearing of Drosophila suzukii for Sterile Insect Technique Application: Evaluation of Two Oviposition Systems
Source: Insects. 2019 Dec 12;10(12):448. doi: 10.3390/insects10120448 (PMC6956338; doi:10.3390/insects10120448)
Supplement: Supplementary file 1 [file insects-10-00448-s001.pdf]

Mass-rearing of *Drosophila suzukii* for Sterile Insect Technique application: Evaluation of two oviposition systems

Fabiana Sassù [1,2]*, Katerina Nikolouli [1,2], Silvana Caravantes [2], Gustavo Taret [3], Rui Pereira [2], Marc J. B.Vreysen [2], Christian Stauffer [1] and Carlos Cáceres [2]

S1. Statistical analyses

R Core Team (2019). R: A language and environment for statistical computing. R Foundation for Statistical Computing, Vienna, Austria. URL https://www.R-project.org/.

Libraries

```
library(Matrix)
library(carDATA)
library(MASS)
library(lme4)
library(nlme)
library(gridExtra)
library(sjmisc)
library(dplyr)
library(emmeans)
library(blmeco)
library(arm)
library(DHARMa)
library(AER)
library(pscl)
library(dispmod)
library(ggplot2)
```

Test the influence of the generations on the two oviposition systems

```
> fit <- lm(ml ~ tec*block, data = mas)
> summary(fit)
Call:
lm(formula = ml ~ tec * block, data = mas)

Residuals:
     Min       1Q   Median       3Q      Max 
-0.48136 -0.16842 -0.00833  0.15500  0.73158 

Coefficients:
               Estimate Std. Error t value Pr(>|t|)    
(Intercept)    0.408333   0.054223   7.531 2.35e-12 ***
tecwax         0.027778   0.076683   0.362    0.718    
block2         0.036667   0.074741   0.491    0.624    
block3         0.060088   0.075667   0.794    0.428    
block4        -0.003333   0.073114  -0.046    0.964    
block5         0.016667   0.079043   0.211    0.833    
tecwax:block2  0.089222   0.105700   0.844    0.400    
tecwax:block3 -0.025146   0.107009  -0.235    0.814    
tecwax:block4  0.058586   0.103399   0.567    0.572    
tecwax:block5  0.019097   0.111783   0.171    0.865    
---
Signif. codes:  0 ‘***’ 0.001 ‘**’ 0.01 ‘*’ 0.05 ‘.’ 0.1 ‘ ’ 1

Residual standard error: 0.23 on 180 degrees of freedom
Multiple R-squared:  0.0388,   Adjusted R-squared:  -0.009261
```

```
F-statistic: 0.8073 on 9 and 180 DF,  p-value: 0.61

> lsmeans(fit, "block")
> lsmeans(fit, pairwise ~ block)
$lsmeans
 block lsmean     SE  df lower.CL upper.CL
 1      0.422 0.0383 180    0.347    0.498
 2      0.503 0.0364 180    0.432    0.575
 3      0.470 0.0373 180    0.396    0.543
 4      0.448 0.0347 180    0.380    0.517
 5      0.448 0.0407 180    0.368    0.529

Results are averaged over the levels of: tec
Confidence level used: 0.95

$contrasts
 contrast  estimate     SE  df t.ratio p.value
 1 - 2    -0.081278 0.0528 180 -1.538  0.5393
 1 - 3    -0.047515 0.0535 180 -0.888  0.9010
 1 - 4    -0.025960 0.0517 180 -0.502  0.9871
 1 - 5    -0.026215 0.0559 180 -0.469  0.9900
 2 - 3     0.033763 0.0521 180  0.648  0.9668
 2 - 4     0.055318 0.0503 180  1.101  0.8060
 2 - 5     0.055063 0.0546 180  1.009  0.8509
 3 - 4     0.021555 0.0509 180  0.423  0.9933
 3 - 5     0.021299 0.0552 180  0.386  0.9953
 4 - 5    -0.000256 0.0534 180 -0.005  1.0000

Results are averaged over the levels of: tec
P value adjustment: tukey method for comparing a family of 5 estimates

> resx<-residuals(fit)
> qqnorm(resx, main = "Normal Q-Q Plot",
       xlab = "Theoretical Quantiles", ylab = "Sample Quantiles",
       plot.it = TRUE, datax = FALSE)
```

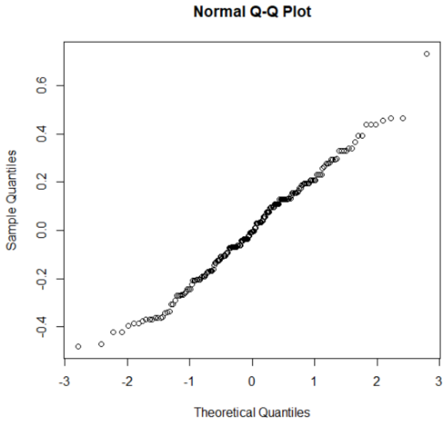


```
> hist(resx)
```

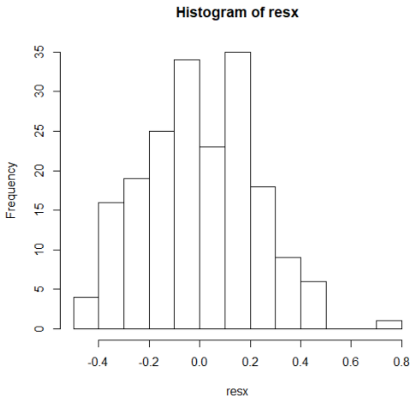


```
> tec_block <- factor(interaction(mas$tec,mas$block))
> tec_block
> kruskal.test (ml ~ tec_block, data=mas)
Kruskal-Wallis chi-squared = 6.0276, df = 9, p-value = 0.7372
```

Egg production (mL)

Summary

```
> group_by(mas, tec) %>%
summarise(
count = n(),
mean = mean(ml, na.rm = TRUE),
sd = sd(ml, na.rm = TRUE),
median = median(ml, na.rm = TRUE),
IQR = IQR(ml, na.rm = TRUE))
 A tibble: 2 x 6
 tec   count  mean    sd median   IQR
  <fct> <int> <dbl> <dbl>  <dbl> <dbl>
1 net      95 0.430 0.213    0.4   0.3
2 wax      95 0.488 0.242    0.5   0.3
```

Descriptive analyses

WAX

```
> mas_join_wax = mas_join[mas_join$tec == "wax",]
> ggplot(data = mas_join_wax,
  aes(x = day_diff, y = ml, color = as.factor(block), size =0.1)) +
  ggtitle("wax treatment ml per experiment by blocks and date") +
geom_line()+ geom_point()
```

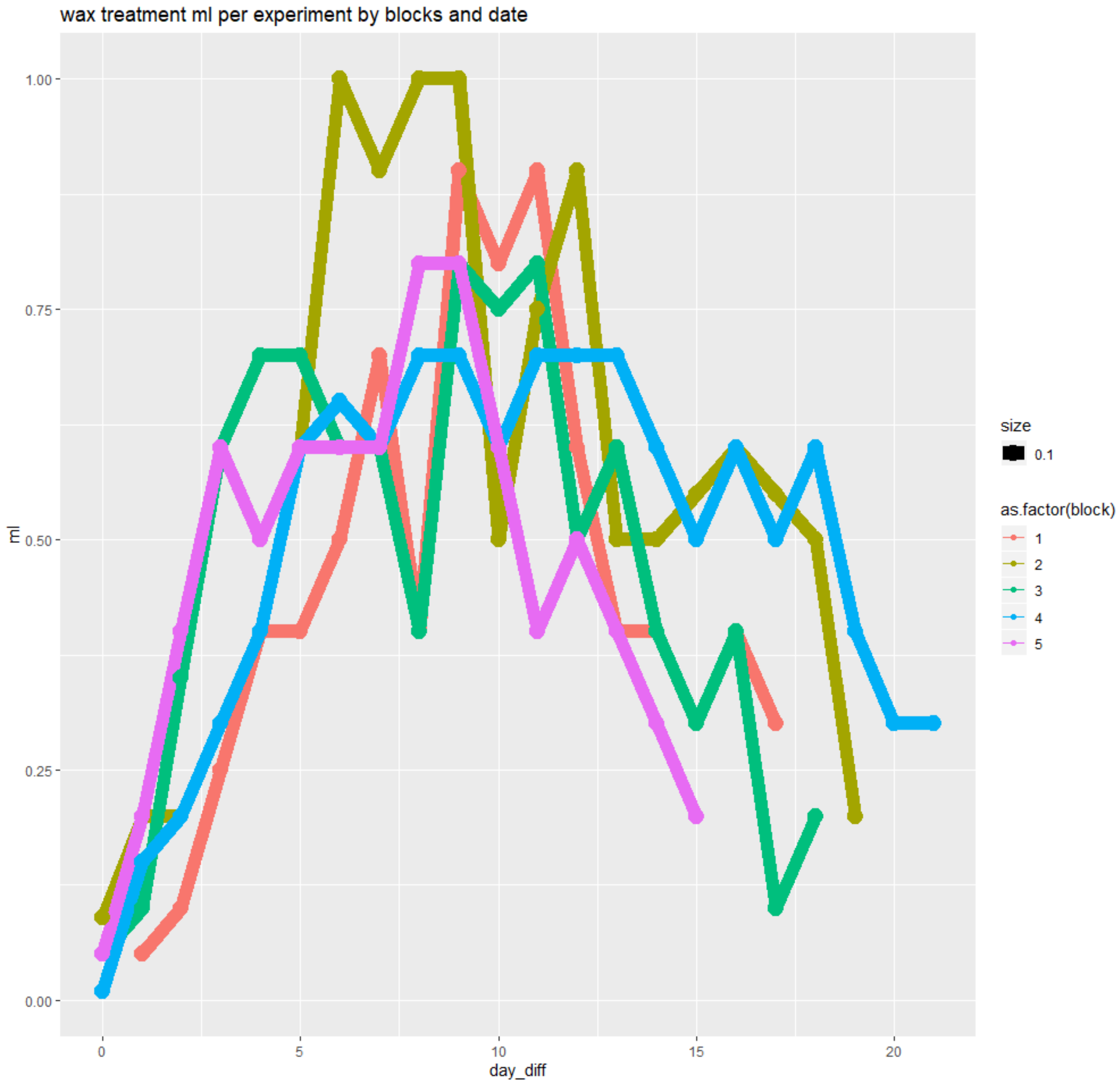

NET

```
> mas_join_net = mas_join[mas_join$tec == "net",]
> ggplot(data = mas_join_net,
       aes(x = day_diff, y = ml, color = as.factor(block), size =0.1)) +
  ggtitle("net treatment ml per experiment by blocks and date") +
  geom_line()+
  geom_point()
```

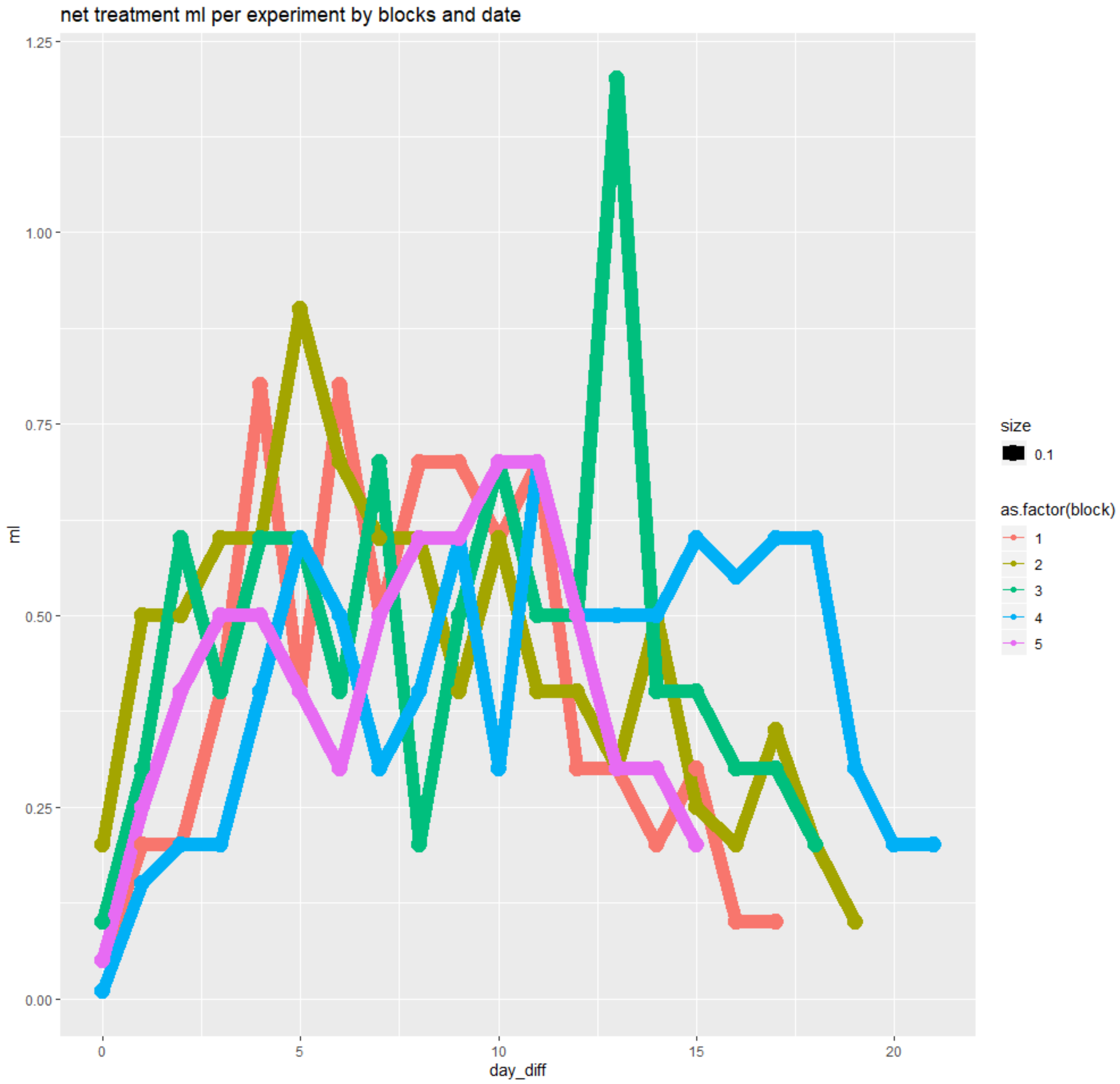

net and wax per all replicas

```
>   boxplot(mas_join$ml~mas_join$tec,   data=   mas_join,   col=c("darkcyan",
"chocolate1"),xlab="Cage type/ All replicates",
+          ylab="egg production (mL)")
```

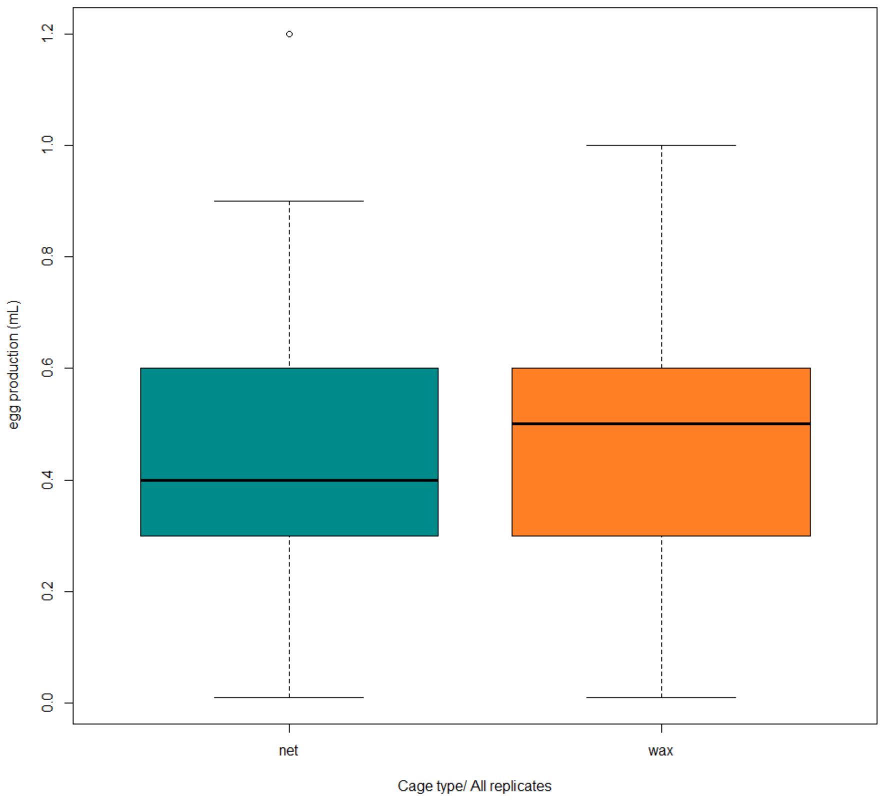


```
Fit model
> model_0 = lme(ml~ as.numeric(day_diff) + tec, random = ~1|block,
+                data = mas_join)
> summary(model_0)
Linear mixed-effects model fit by REML
 Data: mas_join
      AIC      BIC   logLik
  2.58034 18.70907 3.70983

Random effects:
 Formula: ~1 | block
         (Intercept)  Residual
StdDev: 5.949349e-06 0.2261127

Fixed effects: ml ~ as.numeric(day_diff) + tec
                         Value  Std.Error  DF   t-value p-value
(Intercept)          0.4051318 0.03528918 182 11.480339  0.0000
as.numeric(day_diff) 0.0027427 0.00292054 182  0.939125  0.3489
tecwax               0.0627142 0.03289623 182  1.906424  0.0582
 Correlation:
                     (Intr) as.(_)
as.numeric(day_diff) -0.754
tecwax               -0.457 -0.009

Standardized Within-Group Residuals:
        Min          Q1         Med          Q3         Max
-2.02485750 -0.69821185 -0.02761661  0.74051879  3.35767288

Number of Observations: 189
Number of Groups: 5
```

Homoscedasticity

```
> plot(model_0)
```

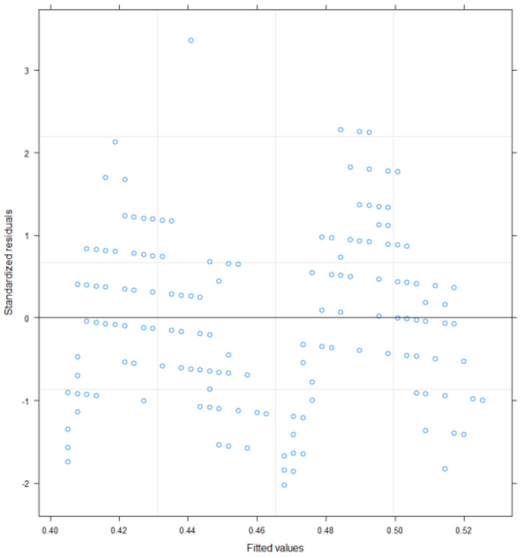


```
> fligner.test(ml~tec, data=mas)
Fligner-Killeen:med chi-squared = 1.1933, df = 1, p-value = 0.2747
```

Normality

```
> res = model_0$residuals
> ggplot(data = resid(model_0))
> hist(, breaks =  20)
> qqnorm(resid(model_0))
> qqline(resid(model_0))
```

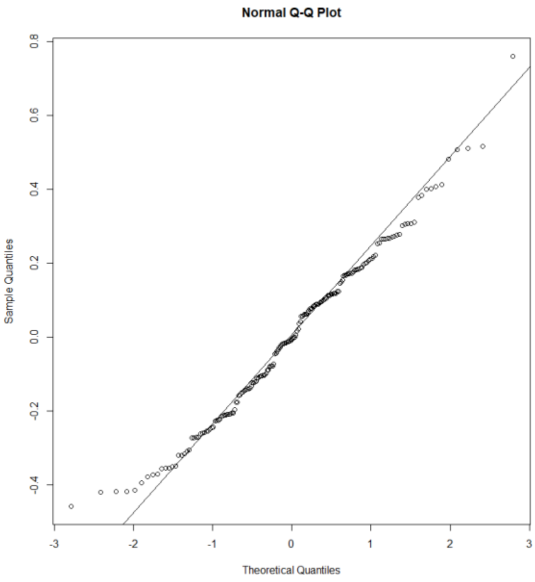


```
> shapiro.test(mas$ml)
W = 0.97614, p-value = 0.002468
```

Non-parametric Wilcox.test

```
> wilcox.test(ml ~ tec, data=mas_join)
W = 3739.5, p-value = 0.05208
```

Egg hatch

Summary

```
> group_by(mas, tec) %>%
  summarise(
  count = n(),
  mean = mean(hatching, na.rm = TRUE),
  sd = sd(hatching, na.rm = TRUE),
  median = median(hatching, na.rm = TRUE),
  IQR = IQR(hatching, na.rm = TRUE))
A tibble: 2 x 6
  tec   count  mean    sd median   IQR
  <fct> <int> <dbl> <dbl>  <dbl> <dbl>
1 net      95  42.7  22.6     38  37.5
2 wax      95  57.3  16.9     58  24
```

Descriptive analyses

WAX

```
> mas_join_wax = mas_join[mas_join$tec == "wax",]
> ggplot(data = mas_join_wax,
+        aes(x = day_diff, y = hatc, color = as.factor(block), size =0.1))
+
+   ggtitle("Wax cage type egg hatch by blocks and date") +
+   geom_line()+
+   geom_point()+
  xlab("Day count") +
  ylab("Egg hatch")
```

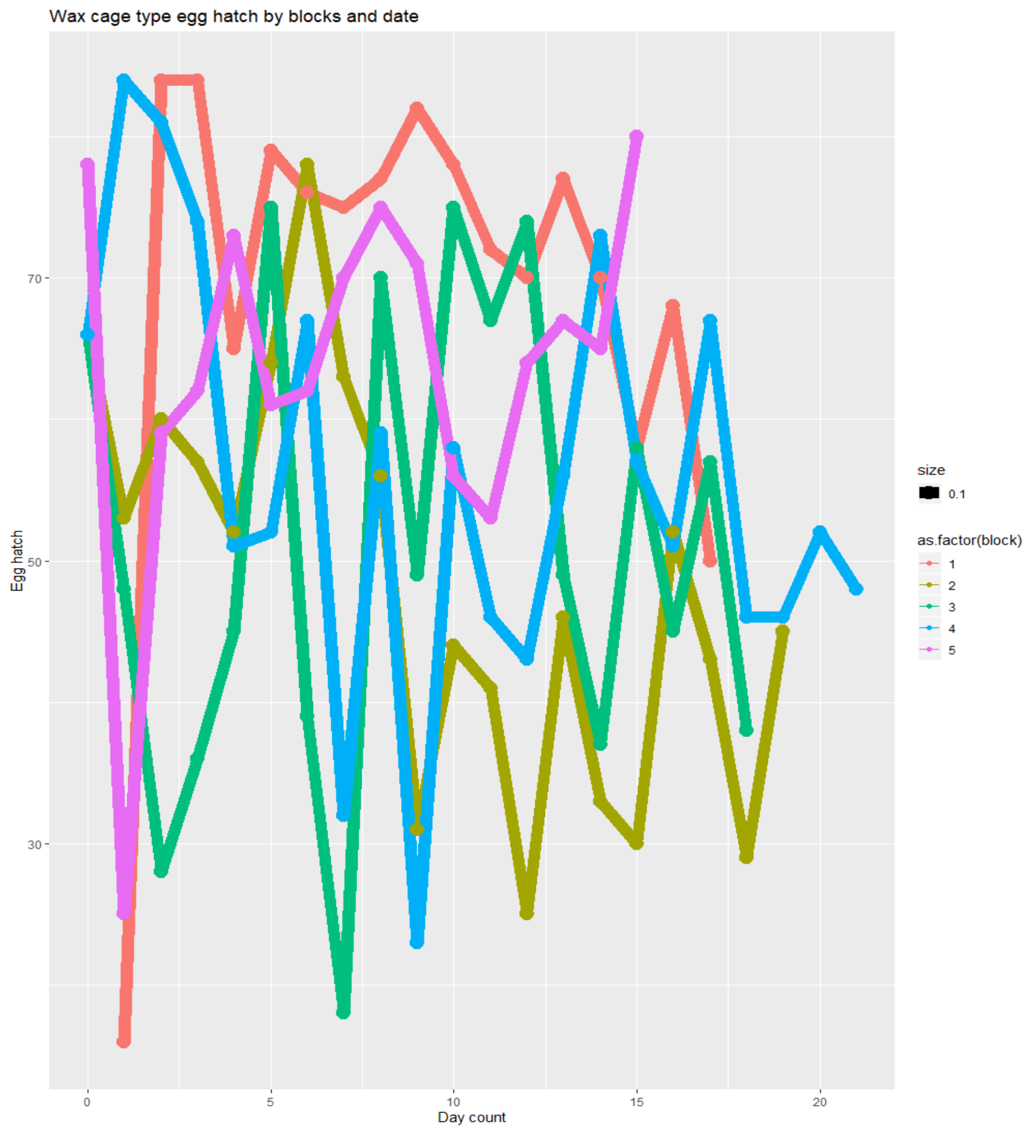

NET

```
> mas_join_net = mas_join[mas_join$tec == "net",]
> ggplot(data = mas_join_net,
+        aes(x = day_diff, y = hatc, color = as.factor(block), size =0.1))
+
+   ggtitle("Net cage type egg hatch by blocks and date") +
+   geom_line()+
+   geom_point()+
  xlab("Day count") +
  ylab("Egg hatch")
```

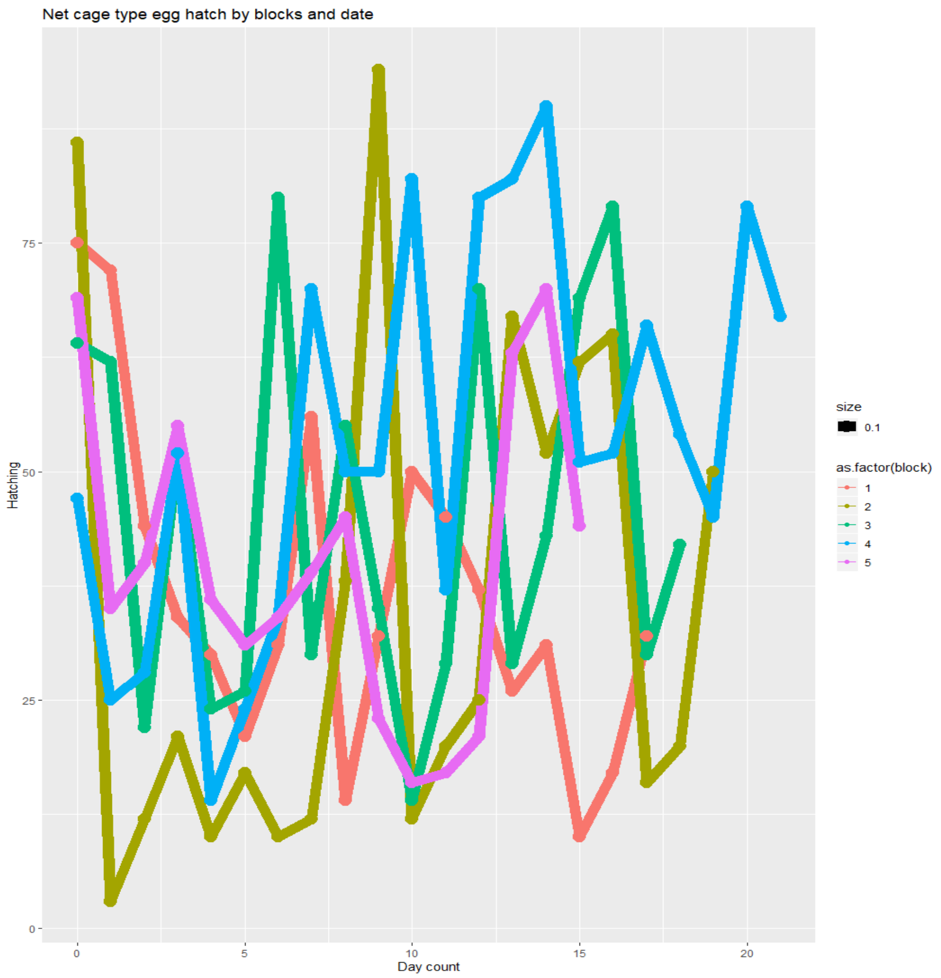


```
net and wax per all replicas
> boxplot(mas_join$hatc~mas_join$tec,  data= mas_join,  col=c("darkcyan",
"chocolate1"),xlab="Cage type",
+          ylab="egg hatched")
```

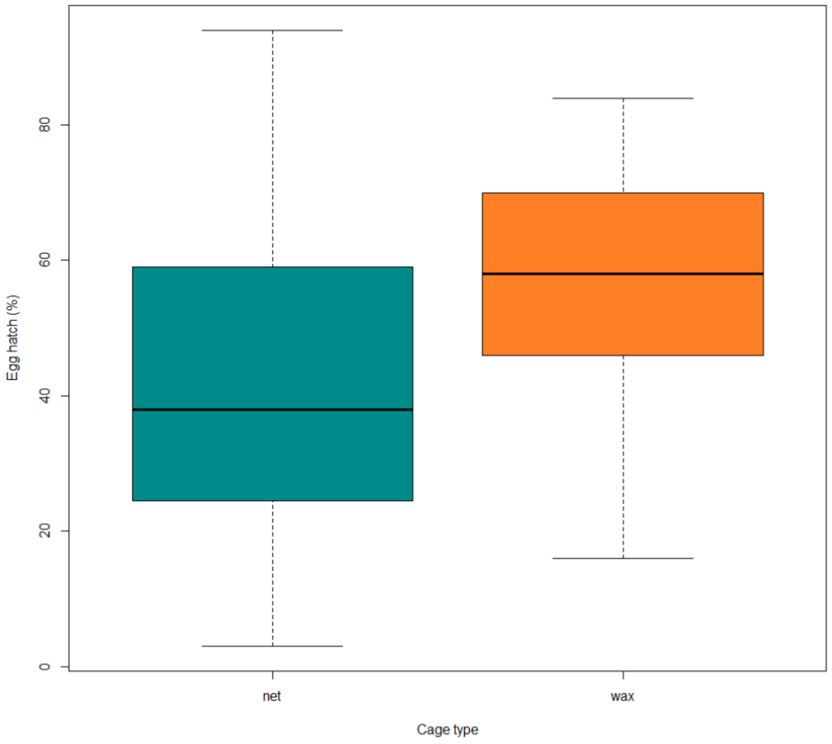


```
Fit model
Model with time
> model_0hatc2 = lme(hatc2~ as.numeric(day_diff) + tec, random = ~1|block,
+                 data = mas_join)
> summary(model_0hatc2)
Linear mixed-effects model fit by REML
 Data: mas_join
       AIC      BIC    logLik
  1662.113 1678.242 -826.0566

Random effects:
 Formula: ~1 | block
        (Intercept) Residual
StdDev:    4.323449 19.35764

Fixed effects: hatc2 ~ as.numeric(day_diff) + tec
                        Value Std.Error  DF   t-value p-value
(Intercept)          42.29832  3.592450 182 11.774227  0.0000
as.numeric(day_diff)  0.00410  0.252493 182  0.016226  0.9871
tecwax               14.88483  2.816373 182  5.285107  0.0000
 Correlation:
                     (Intr) as.(_)
as.numeric(day_diff) -0.635
tecwax               -0.384 -0.008

Standardized Within-Group Residuals:
        Min          Q1         Med          Q3         Max
-2.22460786 -0.73307599  0.01632077  0.69024556  2.95388646

Number of Observations: 189
Number of Groups: 5

 -> Effect of time not significant
 Fit model for hatching without time
```

```
> model_4hatc = lme(hatc~ tec,
+                   random = ~1|block,
+                   data = mas_join)
> summary(model_4hatc)
Linear mixed-effects model fit by REML
 Data: mas_join
        AIC       BIC   logLik
  -63.13778 -50.21334 35.56889

Random effects:
 Formula: ~1 | block
        (Intercept)  Residual
StdDev:  0.04320636 0.1930527

Fixed effects: hatc ~ tec
                Value  Std.Error  DF   t-value p-value
(Intercept) 0.4233539 0.02769677 183 15.285316       0
tecwax      0.1488525 0.02808654 183  5.299779       0
 Correlation:
       (Intr)
tecwax -0.504

Standardized Within-Group Residuals:
       Min         Q1        Med         Q3        Max
-2.2324695 -0.7362335  0.0156079  0.6903789  2.9622248

Number of Observations: 189
Number of Groups: 5

> lsmeans(model_4hatc, pairwise ~ tec)  comparisons of groups
$lsmeans
 tec lsmean     SE df lower.CL upper.CL
 net  0.423 0.0277  4    0.346    0.500
 wax  0.572 0.0278  4    0.495    0.649

d.f. method: containment
Confidence level used: 0.95

$contrasts
 contrast  estimate     SE  df t.ratio p.value
 net - wax   -0.149 0.0281 183 -5.300  <.0001

> comp_ml <- emmeans(model_4hatc, ~tec)
> CLD(comp_ml)
 tec emmean     SE df lower.CL upper.CL .group
 net  0.423 0.0277  4    0.346    0.500  1
 wax  0.572 0.0278  4    0.495    0.649   2

d.f. method: containment
Confidence level used: 0.95
significance level used: alpha = 0.05
> data_pedicted = mas_join
> data_pedicted$predictions = predict(model_4hatc, newdata = mas_join)
>  df_means_in_time_pred  =  aggregate(data_pedicted$predictions,  by  =
list(data_pedicted$day_diff,data_pedicted$tec ), FUN = mean)
> ggplot(data = df_means_in_time_pred,
+         aes(x = day_diff, y = predictions, color = as.factor(tec), size
=0.1)) +
+   ggtitle ("Egg hatch per cage by blocks and date") +
```

```
+   geom_line() +
+   geom_line(aes(day_diff,  hatc, colour=tec), df_means_in_time)
```

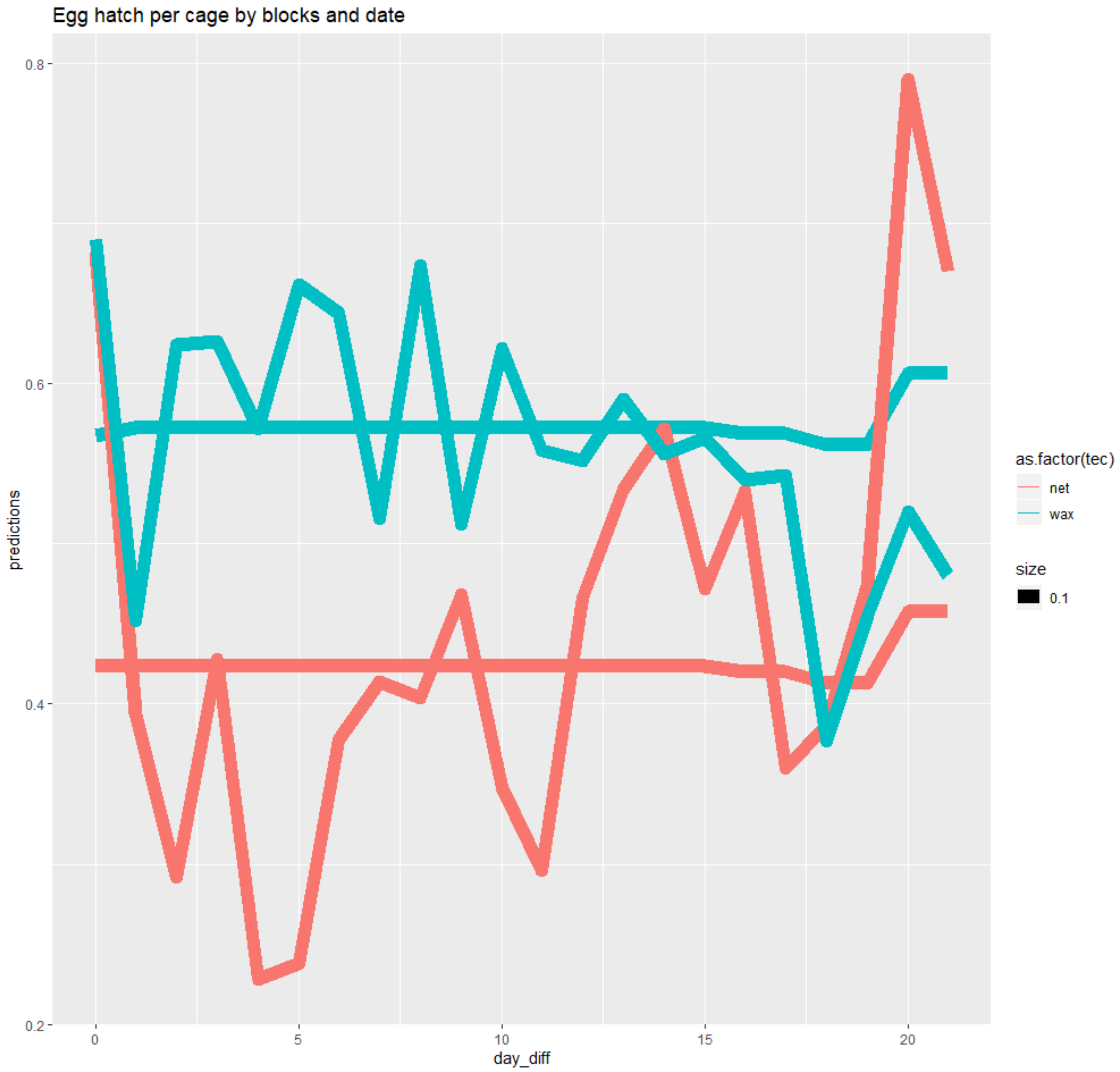

Homoscedasticity

```
> plot(model_4hatc)
```

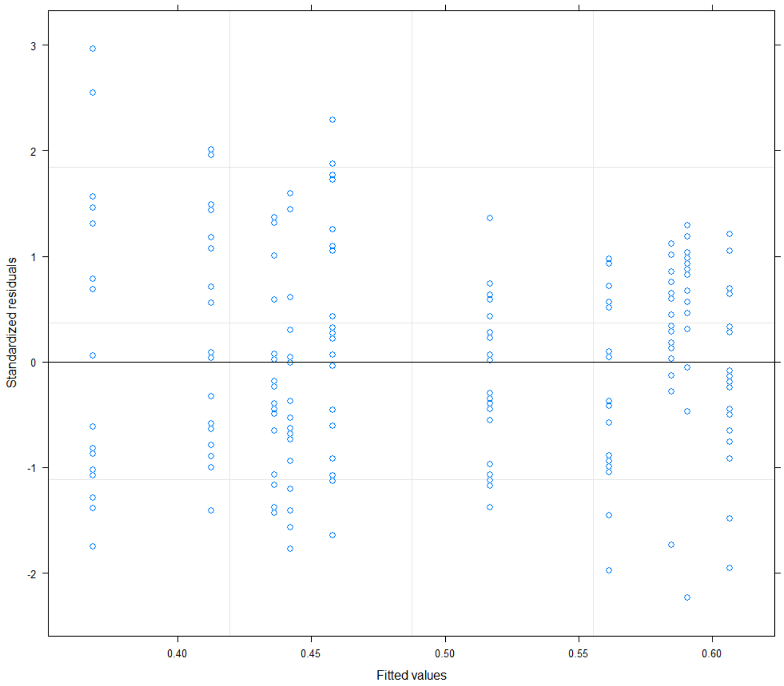


```
> intervals(model_4hatc, which = "fixed")
Approximate 95% confidence intervals

 Fixed effects:
                  lower      est.     upper
(Intercept) 0.36870782 0.4233539 0.4779999
tecwax      0.09343738 0.1488525 0.2042676
attr(,"label")
[1] "Fixed effects:"
> Normality
> class(TONES model_4hatc I$residuals)
> res = model_4hatc$residuals
> ggplot(data = resid(model_4hatc))
> hist(, breaks =  20)
> qqnorm(resid(model_4hatc))
> qqline(resid(model_4hatc))
> qqplot(res[,1])
> qqplot(model_4hatc$residuals)
```

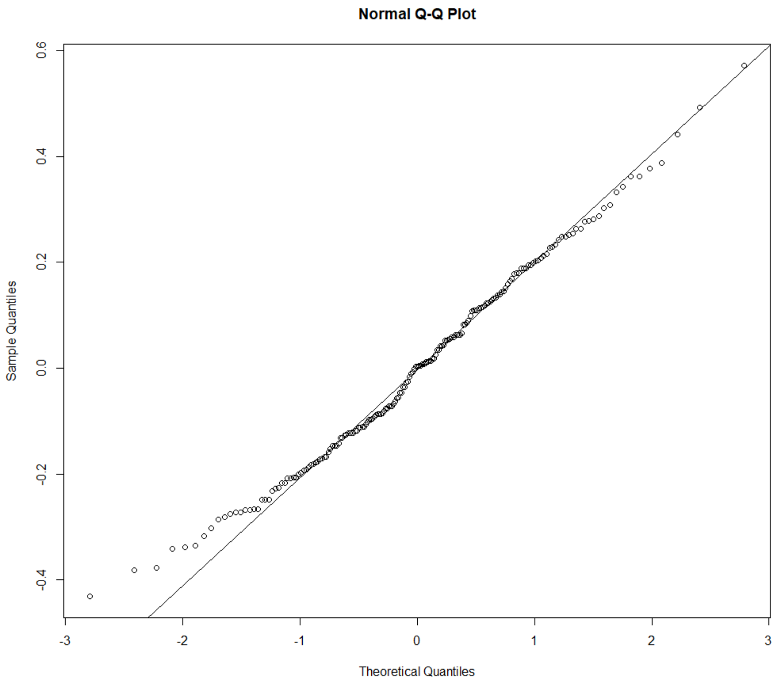


```
> shapiro.test(model_4hatc$residuals)
Shapiro-Wilk normality test
data:  model_4hatc$residuals
W = 0.99038, p-value = 0.01422

> kruskal.test (hatc2 ~ tec, data=mas_join)
Kruskal-Wallis chi-squared = 23.513, df = 1, p-value = 1.24e-06
```

Egg to pupae time (Larval Development)

Summary

```
> group_by(mas, tec) %>%
  summarise(
    count = n(),
    mean = mean(larva.develop, na.rm = TRUE),
    sd = sd(larva.develop, na.rm = TRUE),
    median = median(larva.develop, na.rm = TRUE),
    IQR = IQR(larva.develop, na.rm = TRUE))
A tibble: 2 x 6
 tec   count  mean    sd median   IQR
  <fct> <int> <dbl> <dbl>  <int> <dbl>
1 net      95  7.47 0.867      7     1
2 wax      95  7.63 0.858      8     1

> shapiro.test(mas$larva.develop)
W = 0.86879, p-value = 1.045e-10
> fligner.test(larva.develop~tec, data=mas)
Fligner-Killeen:med chi-squared = 0.027668, df = 1, p-value = 0.8679
> wilcox.test(larva.develop ~ tec, data=mas)
W = 2883.5, p-value = 0.1538
```

Pupa Recovery

Summary

```
> group_by(mas_join, tec) %>%
+   summarise(
```

```
+     count = n(),
+     mean = mean(pupae_prc_hatching, na.rm = TRUE),
+     sd = sd(pupae_prc_hatching, na.rm = TRUE),
+     median = median(pupae_prc_hatching, na.rm = TRUE),
+     IQR = IQR(pupae_prc_hatching, na.rm = TRUE))
A tibble: 2 x 6
  tec   count  mean    sd median   IQR
  <fct> <int> <dbl> <dbl>  <dbl> <dbl>
1 net      95  56.5  21.7   58.6  33.1
2 wax      94  63.5  17.1   66.7  23.2
```

Descriptive analyses

WAX

```
> mas_join_wax = mas_join[mas_join$tec == "wax",]
> ggplot(data = mas_join_wax,
+        aes(x = day_diff, y = pupae, color = as.factor(block), size =0.1))
+   ggtitle("Wax cage pupae recovery by blocks and date") +
+   geom_line()+
+   geom_point()
```

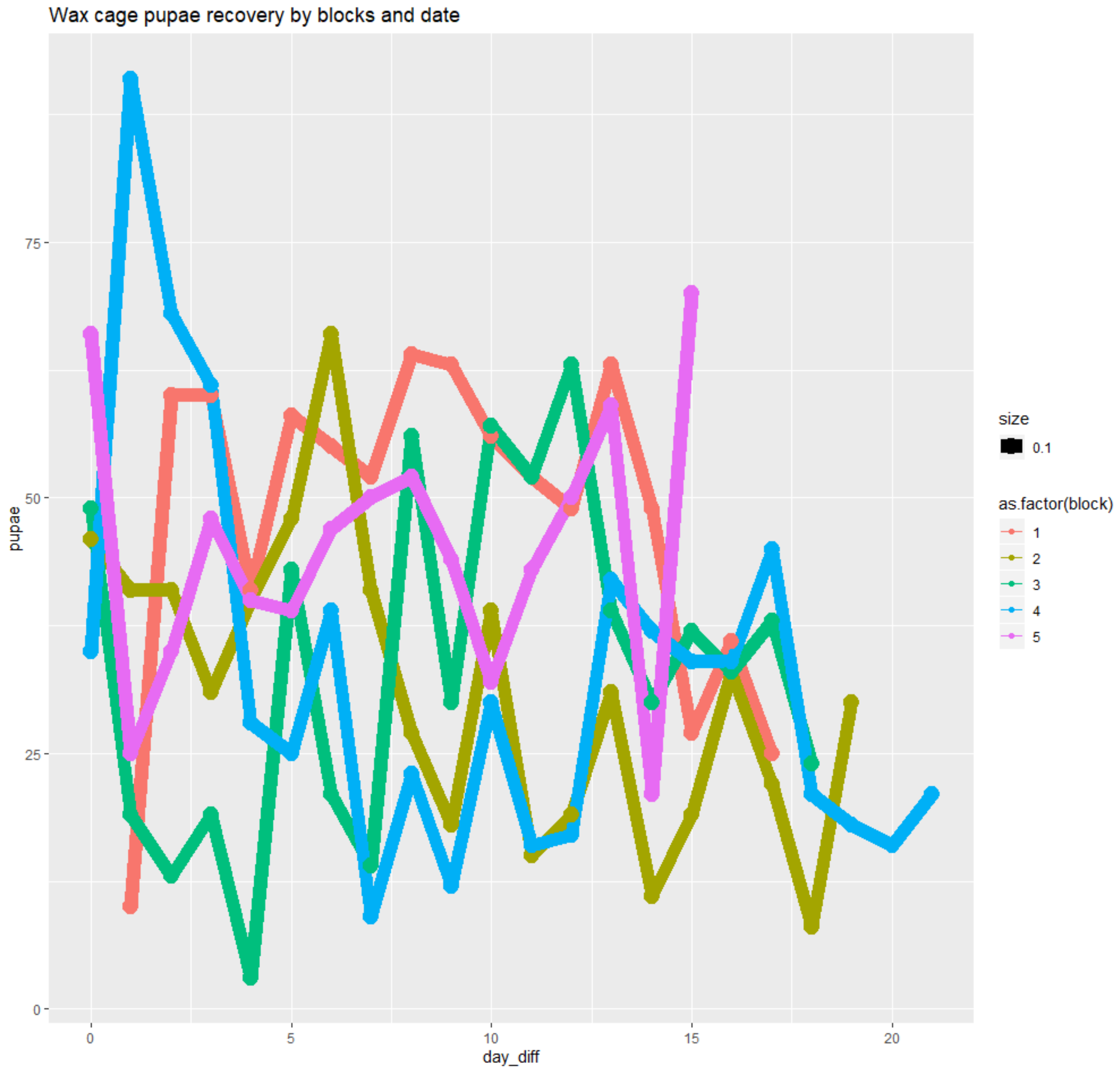

NET

```
> mas_join_net = mas_join[mas_join$tec == "net",]
> ggplot(data = mas_join_net,
```

```
+       aes(x = day_diff, y = pupae_prc_hatching, color = as.factor(block),
size =0.1)) +
+   ggtitle("Net cage pupae recovery by blocks and date") +
+   geom_line()+
+   geom_point()
```

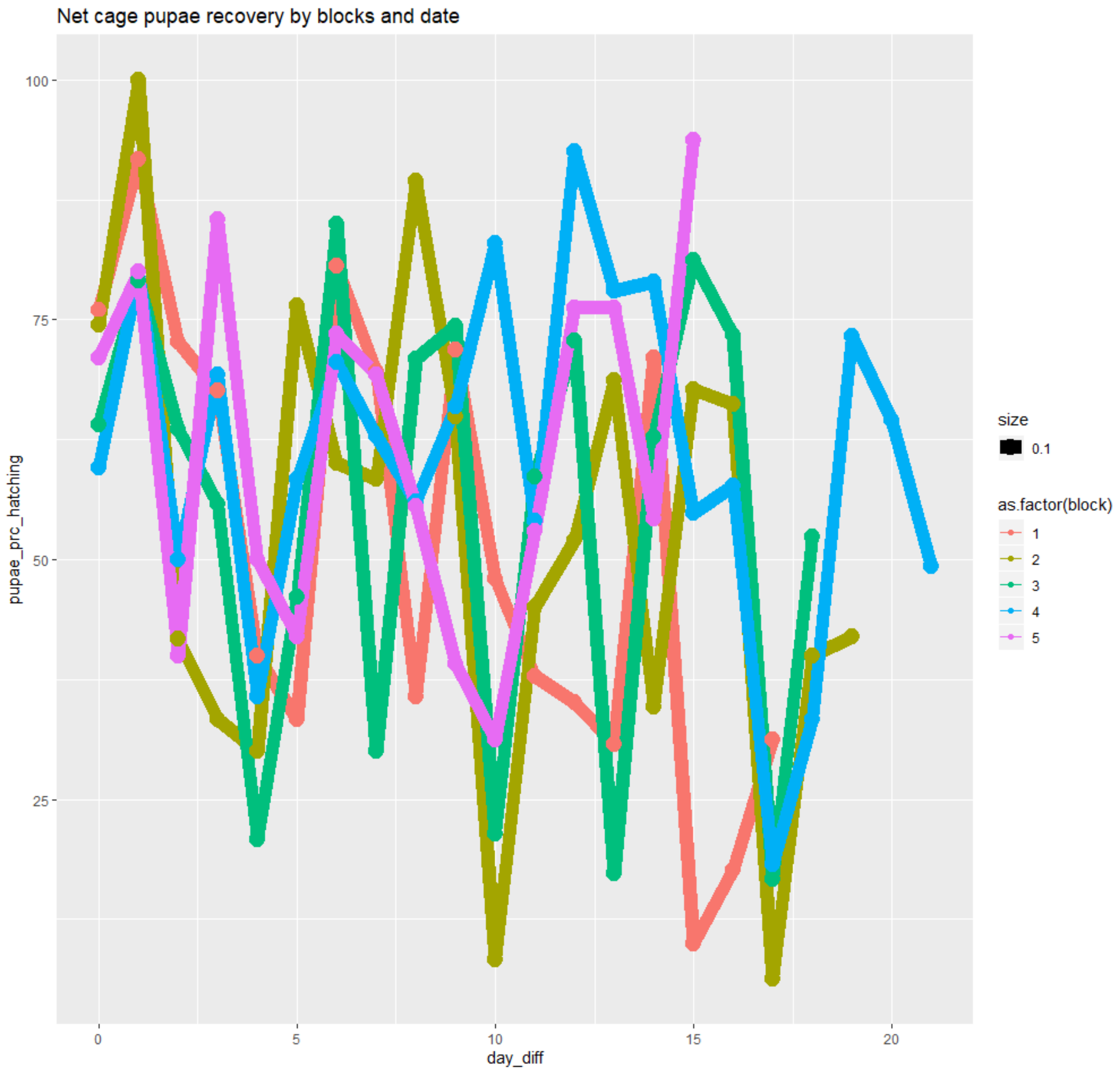

net and wax per all replicas

```
>    boxplot(mas_join$pupae_prc_hatching~mas_join$tec,    data=    mas_join,
col=c("darkcyan", "chocolate1"),xlab="Cage type",
+          ylab="Pupae recover (%)")
```

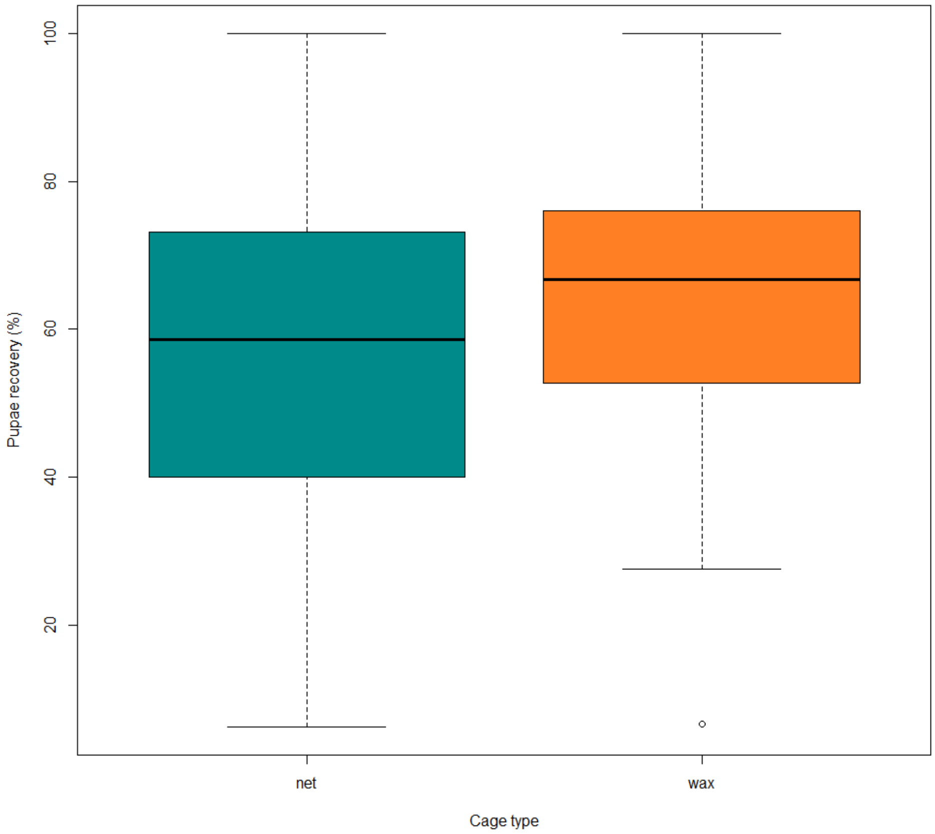


```
Fit model
> model_0pup = lme(pupae_prc_hatching~ as.numeric(day_diff) + tec, random =
~1|block, data = mas_join)
> summary(model_0pup)
Linear mixed-effects model fit by REML
 Data: mas_join
       AIC      BIC    logLik
  1651.469 1667.598 -820.7345

Random effects:
 Formula: ~1 | block
        (Intercept) Residual
StdDev: 0.001252427 19.02539

Fixed effects: pupae_prc_hatching ~ as.numeric(day_diff) + tec
                        Value Std.Error  DF   t-value p-value
(Intercept)          63.97564 2.9692740 182 21.545886  0.0000
as.numeric(day_diff) -0.82645 0.2457375 182 -3.363126  0.0009
tecwax                7.15049 2.7679282 182  2.583335  0.0106
 Correlation:
                     (Intr) as.(_)
as.numeric(day_diff) -0.754
tecwax               -0.457 -0.009

Standardized Within-Group Residuals:
       Min         Q1        Med         Q3        Max
-3.2141438 -0.6791105  0.1636694  0.7609182  2.2165668

Number of Observations: 189
```

```
Number of Groups: 5
 Best fitted model including days
> model_3pup = lme(pupae_prc_hatching~ poly(as.numeric(day_diff),1) + tec,
+                  random = ~1|block,
+                  data = mas_join,
+                  method = "ML")
> summary(model_3pup)
Linear mixed-effects model fit by maximum likelihood
 Data: mas_join
       AIC      BIC    logLik
  1656.837 1673.046 -823.4187

Random effects:
 Formula: ~1 | block
        (Intercept) Residual
StdDev: 0.001087222 18.87379

Fixed effects: pupae_prc_hatching ~ poly(as.numeric(day_diff), 1) + tec
                                  Value Std.Error  DF   t-value p-value
(Intercept)                    56.41082  1.952000 182 28.898986  0.0000
poly(as.numeric(day_diff), 1) -63.98716 19.026095 182 -3.363126  0.0009
tecwax                          7.15049  2.767928 182  2.583335  0.0106
 Correlation:
                              (Intr) p(.(_1
poly(as.numeric(day_diff), 1)  0.006
tecwax                        -0.705 -0.009

Standardized Within-Group Residuals:
       Min         Q1        Med         Q3        Max
-3.2399606 -0.6845652  0.1649840  0.7670301  2.2343708

Number of Observations: 189
Number of Groups: 5
> data_pedicted = mas_join
> data_pedicted$predictions = predict(model_3pup)
> ggplot(data = data_pedicted,
+         aes(x = day_diff, y = predictions, color = as.factor(tec), size
=0.1)) +
+   ggtitle("Pupae recovery by treatment and date") +
+   geom_line() +
+          geom_line(aes(day_diff,      pupae_prc_hatching,   colour=tec),
df_means_in_time)
```

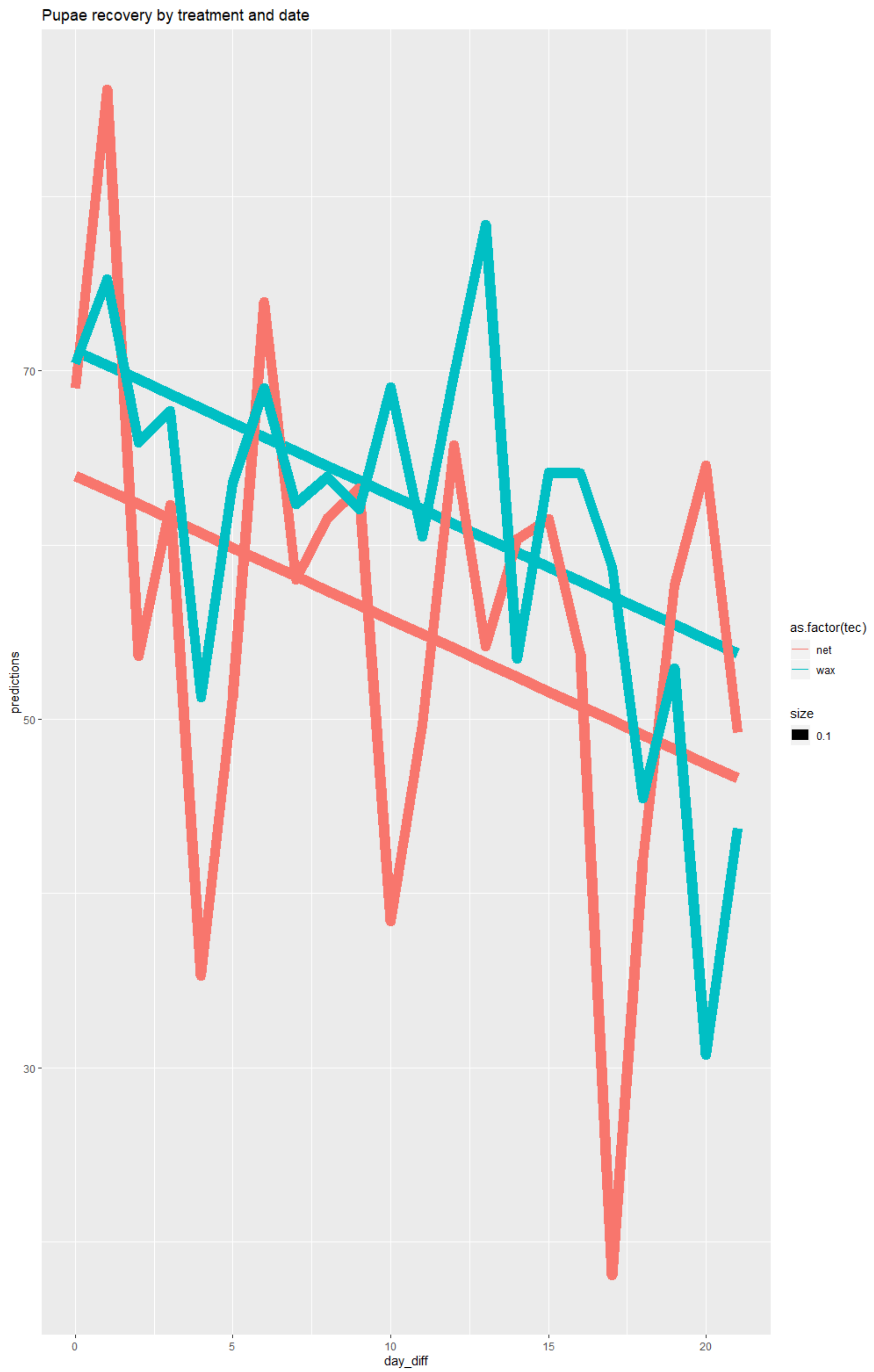


```
> lsmeans(model_3pup, pairwise ~ tec)
$lsmeans
 tec lsmean   SE df lower.CL upper.CL
 net    56.4 1.95  4     51.0     61.8
 wax    63.6 1.96  4     58.1     69.0

d.f. method: containment
```

```
Confidence level used: 0.95

$contrasts
 contrast  estimate   SE  df t.ratio p.value
 net - wax    -7.15 2.77 182 -2.583  0.0106

> comp_ml <- emmeans(model_3pup, ~tec)
> CLD(comp_ml)
 tec emmean   SE df lower.CL upper.CL .group
 net    56.4 1.95  4     51.0     61.8  1
 wax    63.6 1.96  4     58.1     69.0   2

d.f. method: containment
Confidence level used: 0.95
significance level used: alpha = 0.05
```

Homoscedasticity

```
> plot(model_3pup)
> intervals(model_3pup, which = "fixed")
Approximate 95% confidence intervals

 Fixed effects:
                                  lower       est.     upper
(Intercept)                   52.590046  56.410817  60.23159
poly(as.numeric(day_diff), 1) -101.228118 -63.987163 -26.74621
tecwax                         1.732648   7.150486  12.56832
attr(,"label")
[1] "Fixed effects:"
```

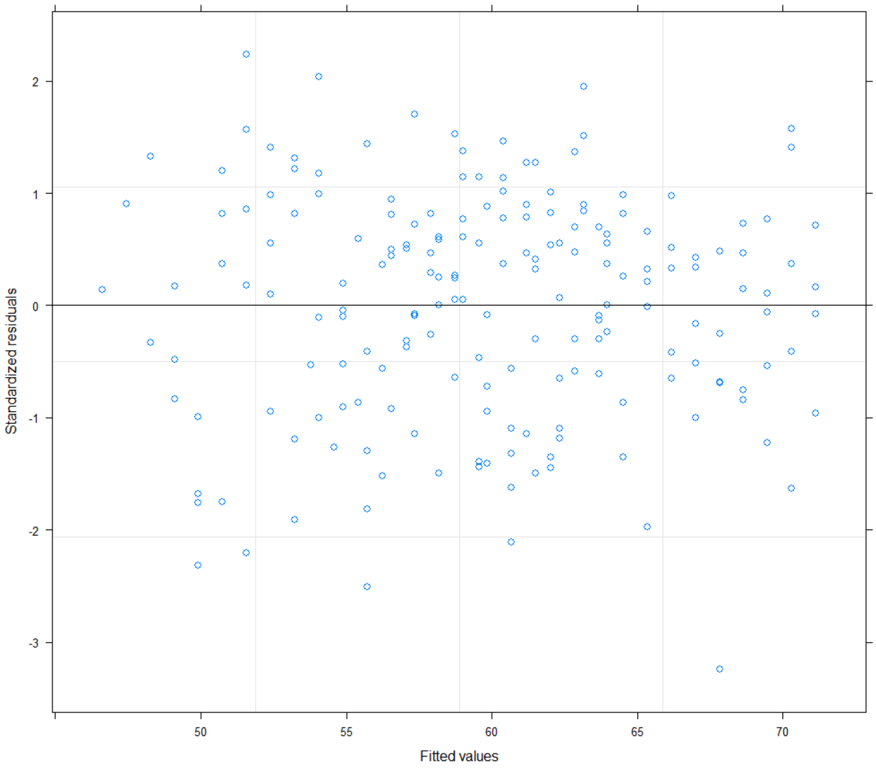

Normality

```
> res = model_3pup$residuals
> ggplot(data = resid(model_3pup))
> hist(, breaks =  20)
> qqnorm(resid(model_3pup))
> qqline(resid(model_3pup))
```

```
> qqplot(res[,1])
> qqplot(model_3pup$residuals)
```

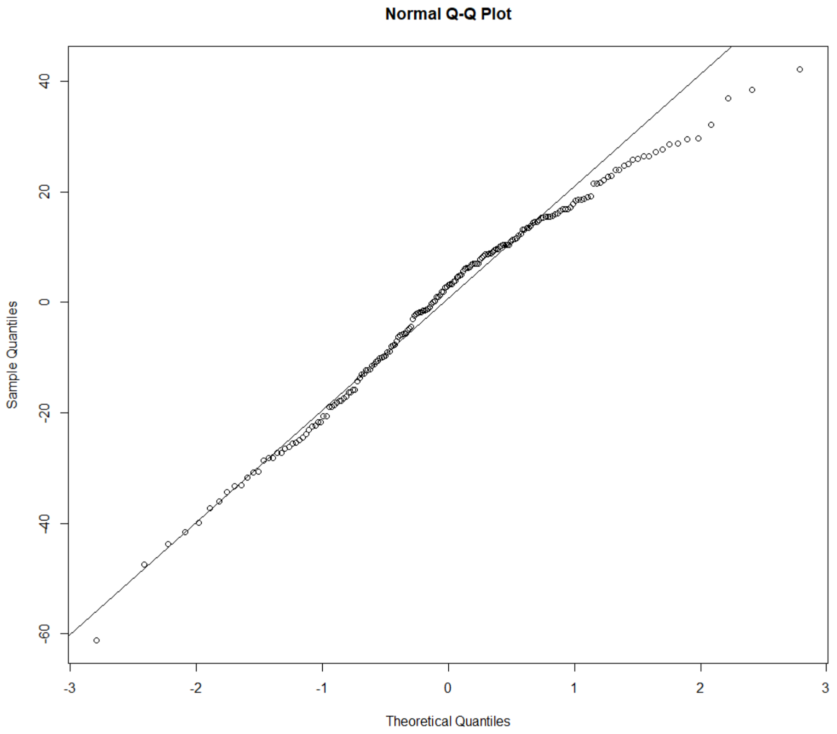


```
> shapiro.test(model_3pup$residuals)
Shapiro-Wilk normality test
data:  model_3pup$residuals
W = 0.98146, p-value = 8.9e-05

> kruskal.test (pupae_prc_hatching ~ tec, data=mas_join)
Kruskal-Wallis chi-squared = 4.538, df = 1, p-value = 0.03315
```

Adult Production

```
Summary
> group_by(mas_join, tec) %>%
+   summarise(
+     count = n(),
+     mean = mean(adult_prc_hatch, na.rm = TRUE),
+     sd = sd(adult_prc_hatch, na.rm = TRUE),
+     median = median(adult_prc_hatch, na.rm = TRUE),
+     IQR = IQR(adult_prc_hatch, na.rm = TRUE))
# A tibble: 2 x 6
  tec   count  mean    sd median   IQR
  <fct> <int> <dbl> <dbl>  <dbl> <dbl>
1 net      95  50.4  21.6   52.4  28.4
2 wax      94  56.9  16.3   59.1  19.2
Descriptive analyses
WAX
> mas_join_wax = mas_join[mas_join$tec == "wax",]
> ggplot(data = mas_join_wax,
+         aes(x = day_diff, y = adult_prc_hatch, color = as.factor(block),
size =0.1)) +
+    ggtitle("wax treatment adult_prc_hatch per experiment by blocks and
date") +
+   geom_line()+
+   geom_point()
```

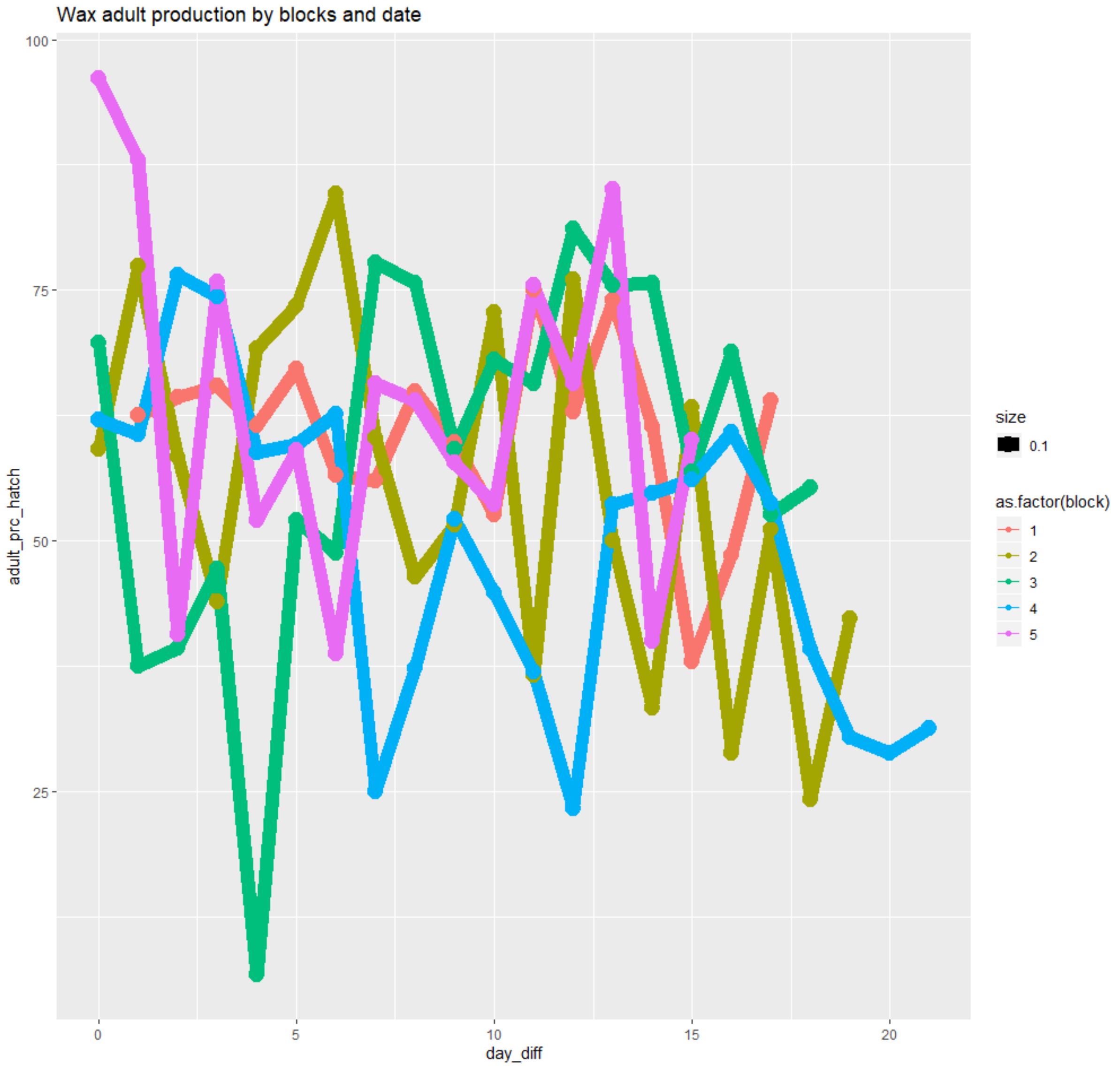

NET

```
> mas_join_net = mas_join[mas_join$tec == "net",]
> ggplot(data = mas_join_net,
+         aes(x = day_diff, y = adult_prc_hatch, color = as.factor(block),
size =0.1)) +
+   ggtitle("Net adult production by blocks and date") +
+   geom_line()+
+   geom_point()
```

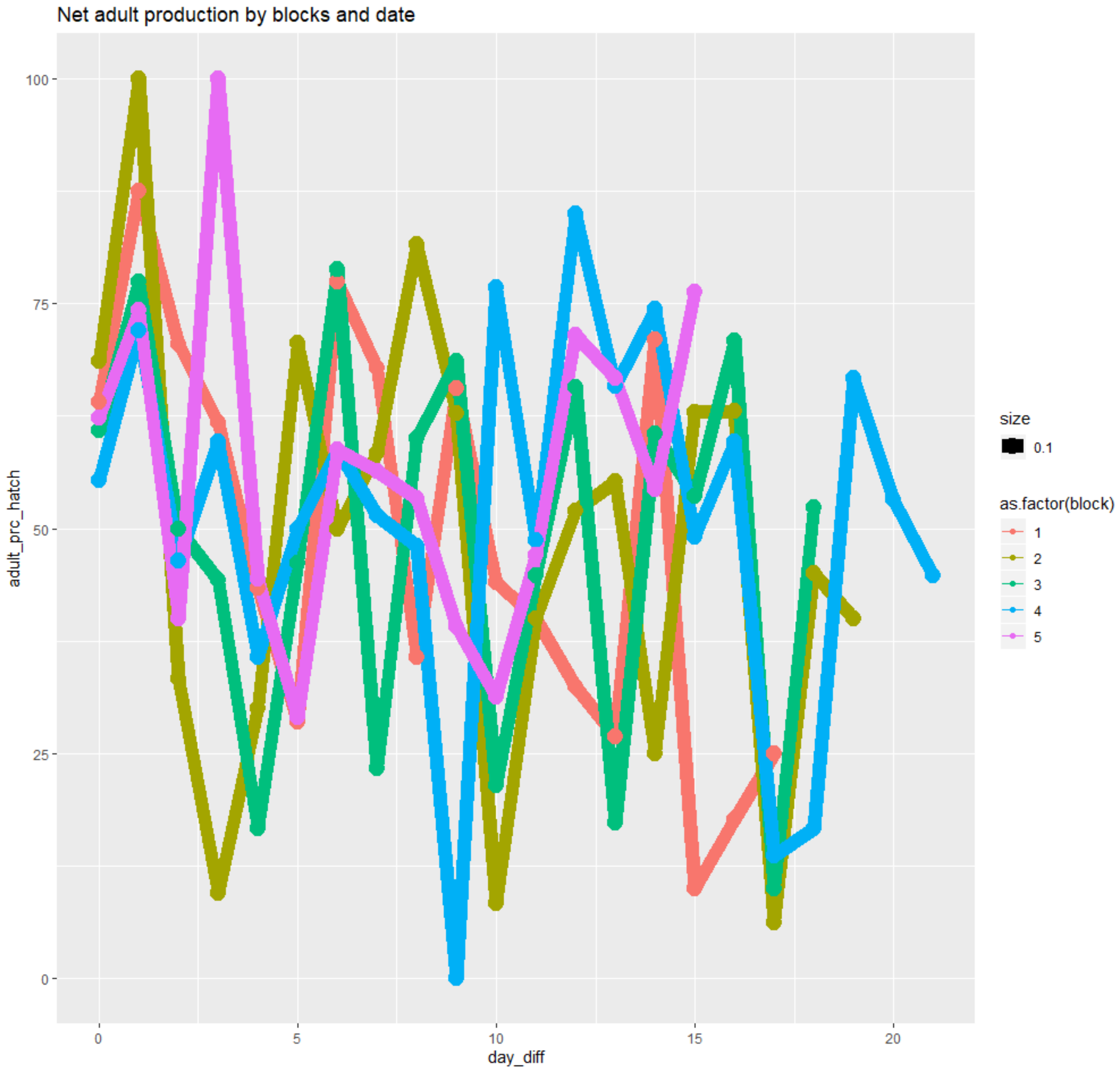

net and wax per all replicas

```
>     boxplot(mas_join$adult_prc_hatch~mas_join$tec,     data=     mas_join,
col=c("darkcyan", "chocolate1"),xlab="Cage type",
+          ylab="Adult production (%)")
```

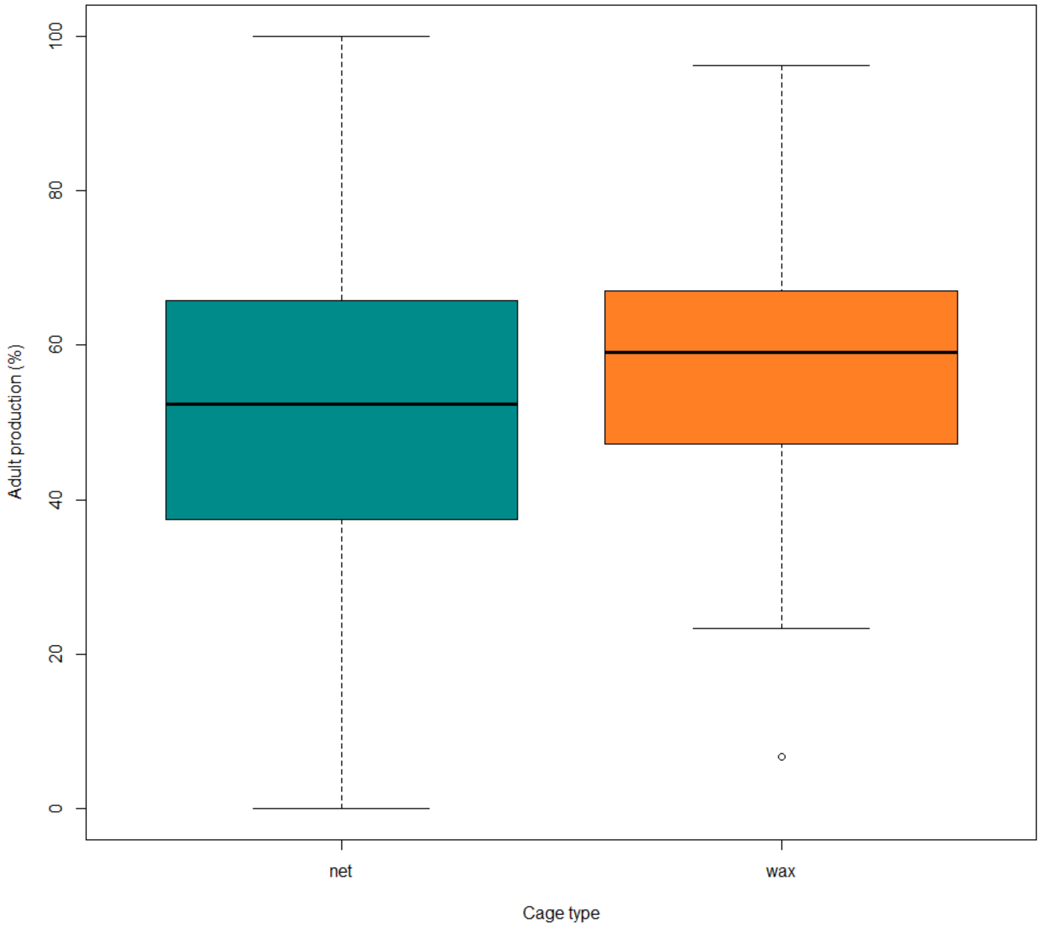


```
Fit Model
> model_0adult_prc_hatch = lme(adult_prc_hatch~ as.numeric(day_diff) + tec,
random = ~1|block,+ data = mas_join)
> summary(model_0adult_prc_hatch)
Linear mixed-effects model fit by REML
 Data: mas_join
       AIC      BIC    logLik
  1641.382 1657.51 -815.6908

Random effects:
 Formula: ~1 | block
        (Intercept) Residual
StdDev: 0.001506003 18.51642

Fixed effects: adult_prc_hatch ~ as.numeric(day_diff) + tec
                        Value Std.Error  DF   t-value p-value
(Intercept)          58.56424 2.8898399 182 20.265565  0.0000
as.numeric(day_diff) -0.89352 0.2391635 182 -3.736012  0.0003
tecwax                6.58462 2.6938805 182  2.444288  0.0155
 Correlation:
                     (Intr) as.(_)
as.numeric(day_diff) -0.754
tecwax               -0.457 -0.009

Standardized Within-Group Residuals:
       Min         Q1        Med         Q3        Max
-2.9651941 -0.6248895  0.1119438  0.7077317  2.3825505
```

```
Number of Observations: 189
Number of Groups: 5

Fit model including days
>          model_3adult_prc_hatch          =          lme(adult_prc_hatch~
poly(as.numeric(day_diff),2) + tec, +random = ~1|block,+ data = mas_join)
> summary(model_3adult_prc_hatch)
Linear mixed-effects model fit by REML
 Data: mas_join
      AIC      BIC    logLik
  1626.941 1646.263 -807.4705

Random effects:
 Formula: ~1 | block
        (Intercept) Residual
StdDev: 0.001375626 18.56319

Fixed effects: adult_prc_hatch ~ poly(as.numeric(day_diff), 2) + tec
                                   Value Std.Error  DF   t-value p-value
(Intercept)                     50.38886  1.904624 181 26.456059  0.0000
poly(as.numeric(day_diff), 2)1 -69.17974 18.563872 181 -3.726579  0.0003
poly(as.numeric(day_diff), 2)2  -4.69937 18.564101 181 -0.253143  0.8004
tecwax                           6.57783  2.700817 181  2.435497  0.0158
 Correlation:
                               (Intr) p(.(_),2)1 p(.(_),2)2
poly(as.numeric(day_diff), 2)1  0.006
poly(as.numeric(day_diff), 2)2 -0.007  0.000
tecwax                         -0.705 -0.009      0.010

Standardized Within-Group Residuals:
        Min          Q1         Med          Q3         Max
-2.95885523 -0.63203011  0.09280197  0.72430598  2.38221337

Number of Observations: 189
Number of Groups: 5
> lsmeans(model_3adult_prc_hatch, pairwise ~ tec)  comparisons of groups
$lsmeans
 tec lsmean   SE df lower.CL upper.CL
 net   50.7 2.37  4     44.2     57.3
 wax   57.3 2.36  4     50.8     63.9

d.f. method: containment
Confidence level used: 0.95

$contrasts
 contrast  estimate  SE  df t.ratio p.value
 net - wax    -6.58 2.7 181 -2.435  0.0158

> comp_ml <- emmeans(model_3adult_prc_hatch, ~tec)
> CLD(comp_ml)
 tec emmean   SE df lower.CL upper.CL .group
 net   50.7 2.37  4     44.2     57.3  1
 wax   57.3 2.36  4     50.8     63.9   2

d.f. method: containment
Confidence level used: 0.95
significance level used: alpha = 0.05
> data_pedicted = mas_join
```

```
> data_pedicted$predictions = predict(model_3adult_prc_hatch, newdata =
mas_join)
> df_means_in_time_pred = aggregate(data_pedicted$predictions, by =
list(data_pedicted$day_diff,data_pedicted$tec ), FUN = mean)

> df_means_in_time = aggregate(mas_join$adult_prc_hatch, by =
list(mas_join$day_diff,mas_join$tec ), FUN = mean)
> names(df_means_in_time) <- c("day_diff", "tec", "adult_prc_hatch")

> ggplot(data = df_means_in_time_pred,
+        aes(x = day_diff, y = predictions, color = as.factor(tec), size
=0.1)) +
+   ggtitle("Fit model for adult production by cage and date") +
+   geom_line() +
+   geom_line(aes(day_diff, adult_prc_hatch, colour=tec), df_means_in_time)
```

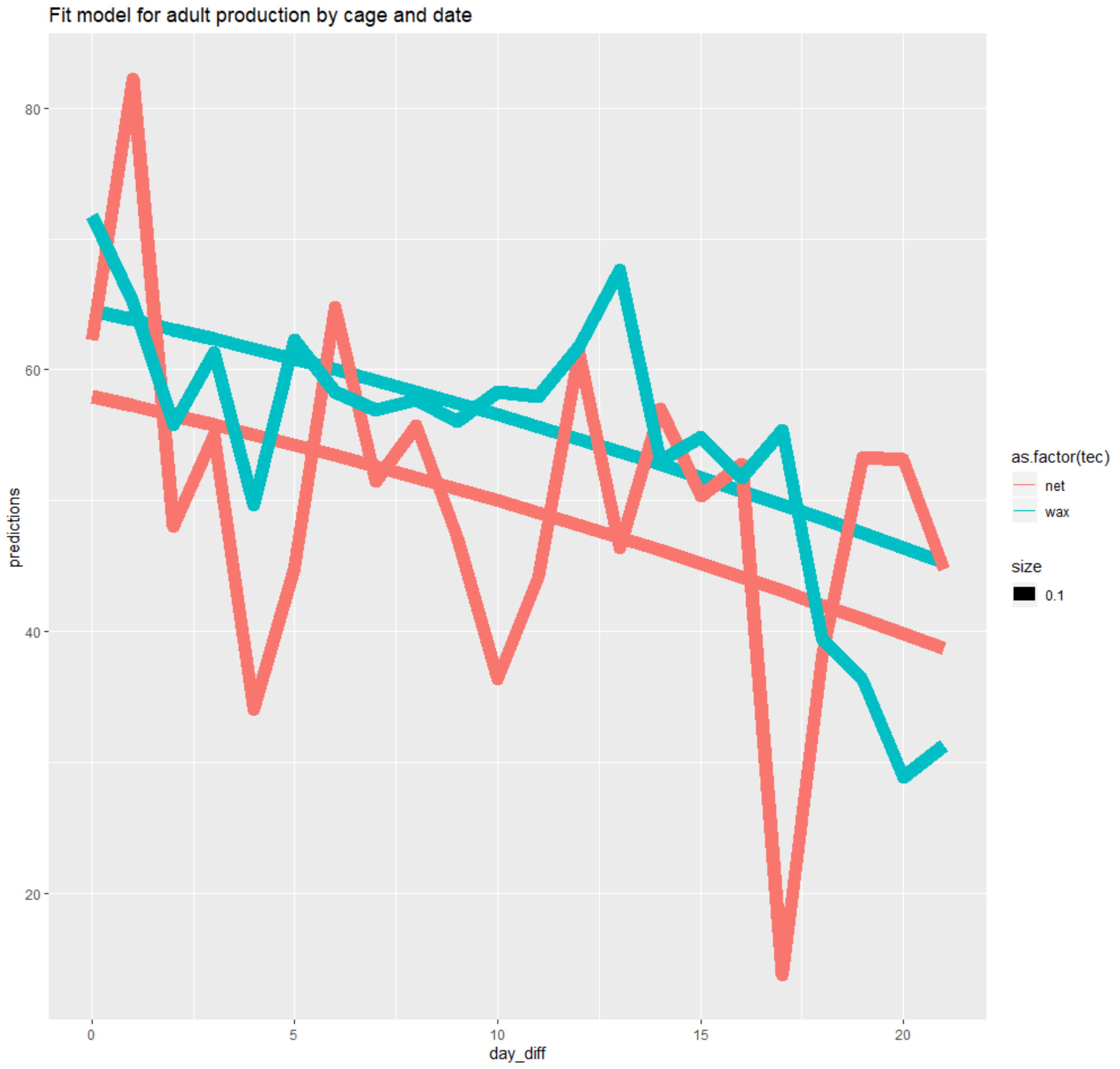


```
> model_3adult_prc_hatch$coefficients
$fixed
                  (Intercept)       poly(as.numeric(day_diff),        2)1
poly(as.numeric(day_diff), 2)2
                    50.388857                -69.179741                 -
4.699373
                       tecwax
                     6.577830
```

```
$random
$random$block
    (Intercept)
1  7.246706e-08
2 -3.785584e-07
3 -5.286917e-08
4 -4.919520e-07
5  8.509125e-07
```

Homoscedasticity

```
> plot(model_3adult_prc_hatch)
> intervals(model_3adult_prc_hatch, which = "fixed")
Approximate 95% confidence intervals

 Fixed effects:
                                     lower        est.     upper
(Intercept)                      46.670715  50.388857  54.10700
poly(as.numeric(day_diff), 2)1 -105.419490 -69.179741 -32.93999
poly(as.numeric(day_diff), 2)2  -40.939568  -4.699373  31.54082
tecwax                            1.305389   6.577830  11.85027
attr(,"label")
[1] "Fixed effects:"
```

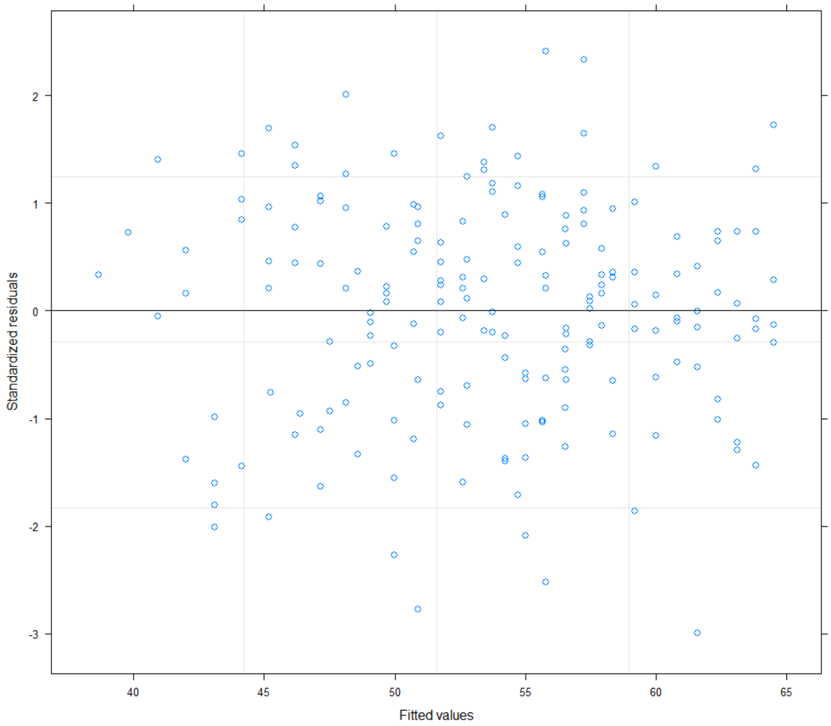

Normality

```
> res = model_3adult_prc_hatch$residuals
> ggplot(data = resid(model_3adult_prc_hatch))
> hist(, breaks =  20)
> qqnorm(resid(model_3adult_prc_hatch))
> qqline(resid(model_3adult_prc_hatch))
> qqplot(res[,1])
> qqplot(model_3adult_prc_hatch$residuals)
```

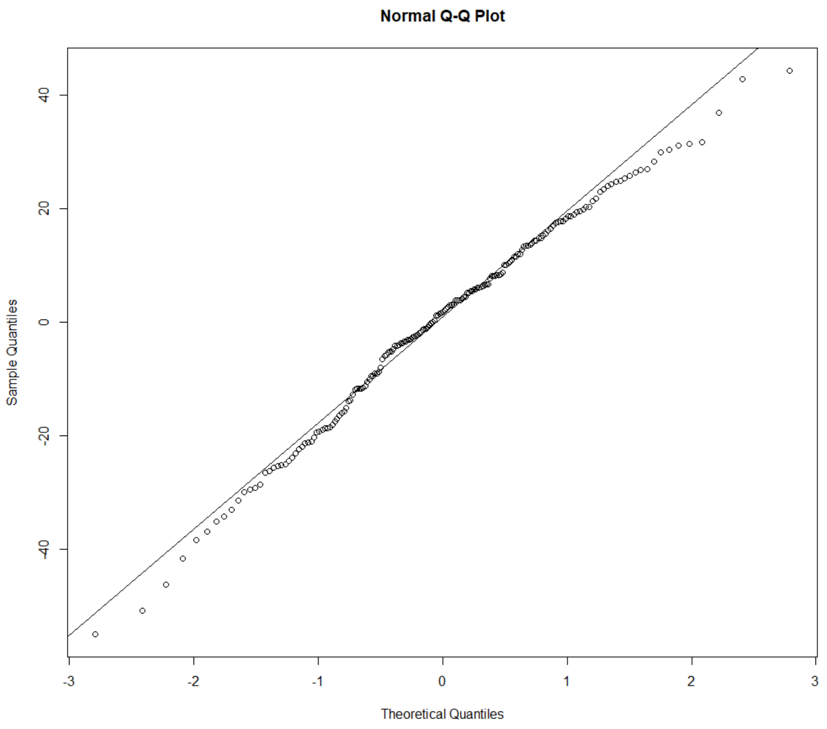


```
> shapiro.test(model_3adult_prc_hatch$residuals)
Shapiro-Wilk normality test

data:  model_3adult_prc_hatch$residuals
W = 0.98611, p-value = 0.001094

> kruskal.test (adult_prc_hatch ~ tec, data=mas_join)
Kruskal-Wallis chi-squared = 4.0692, df = 1, p-value = 0.04367
```

Qc parameters

Emergence

Summary

```
> group_by(mas, tec) %>%
  summarise(
    count = n(),
    mean = mean(emerge_prc, na.rm = TRUE),
    sd = sd(emerge_prc, na.rm = TRUE),
    median = median(emerge_prc, na.rm = TRUE),
    IQR = IQR(emerge_prc, na.rm = TRUE))
A tibble: 2 x 6
  tec   count  mean    sd median   IQR
  <fct> <int> <dbl> <dbl>  <dbl> <dbl>
1 net      95  74.9  21.2     80    30
2 wax      95  85.5  12.5     90    20
```

Descriptive analyses

WAX

```
> ggplot(data = mas2_join_net,
+        aes(x = daycount, y = eme2, color = as.factor(block), size =0.1))
+   ggtitle()+
+   geom_point()+ xlab("Day count") +
+   ylab("Emergence (%)")
Error in labs(title = label, subtitle = subtitle) :
  argument "label" is missing, with no default
```

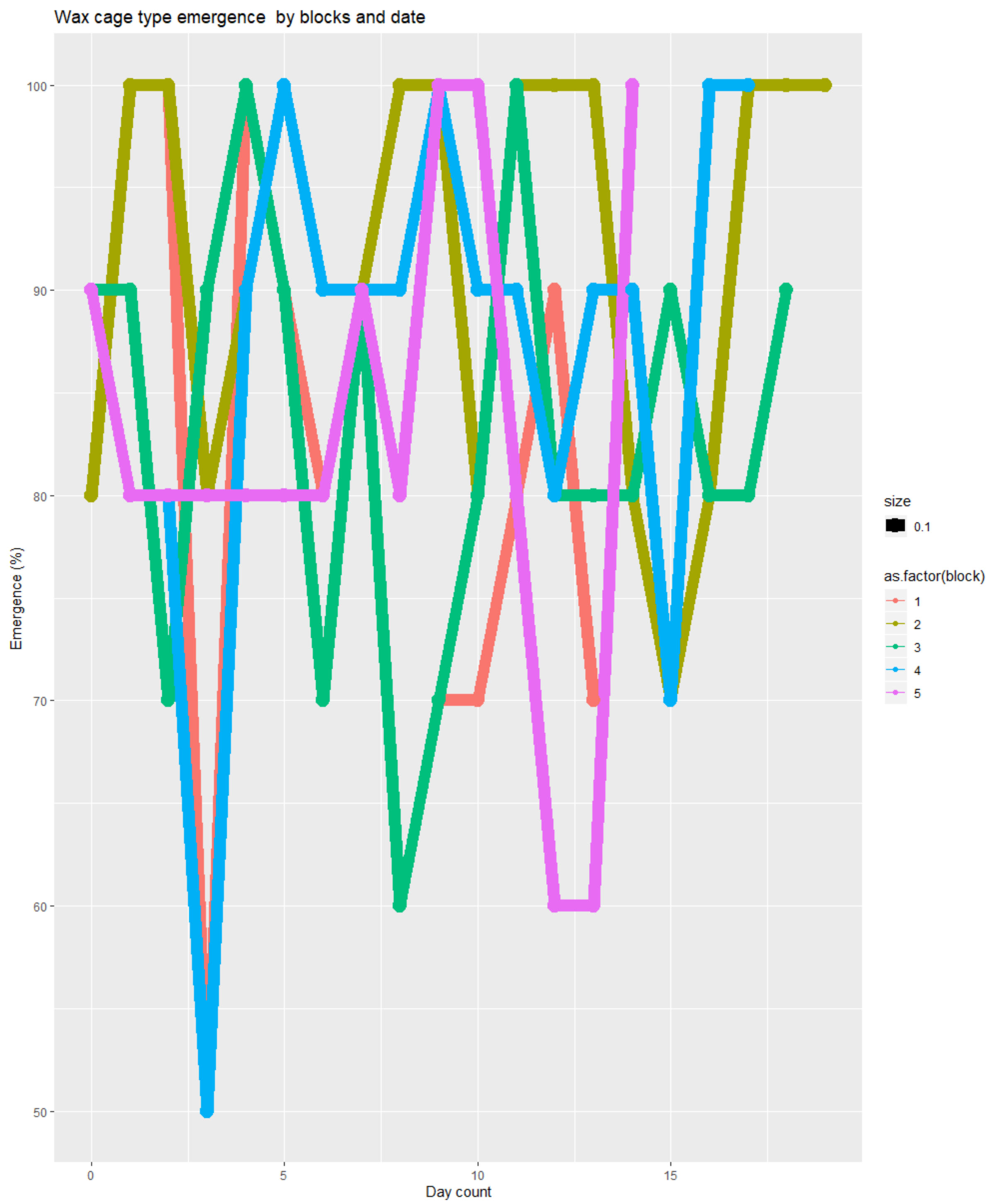

NET

```
> ggplot(data = mas2_join_net,
+        aes(x = daycount, y = eme2, color = as.factor(block), size =0.1))
+
+   ggtitle("Net cage type emergence  by blocks and date") +
+   geom_line()+
+   geom_point()+ xlab("Day count") +
+   ylab("Emergence (%)")
```

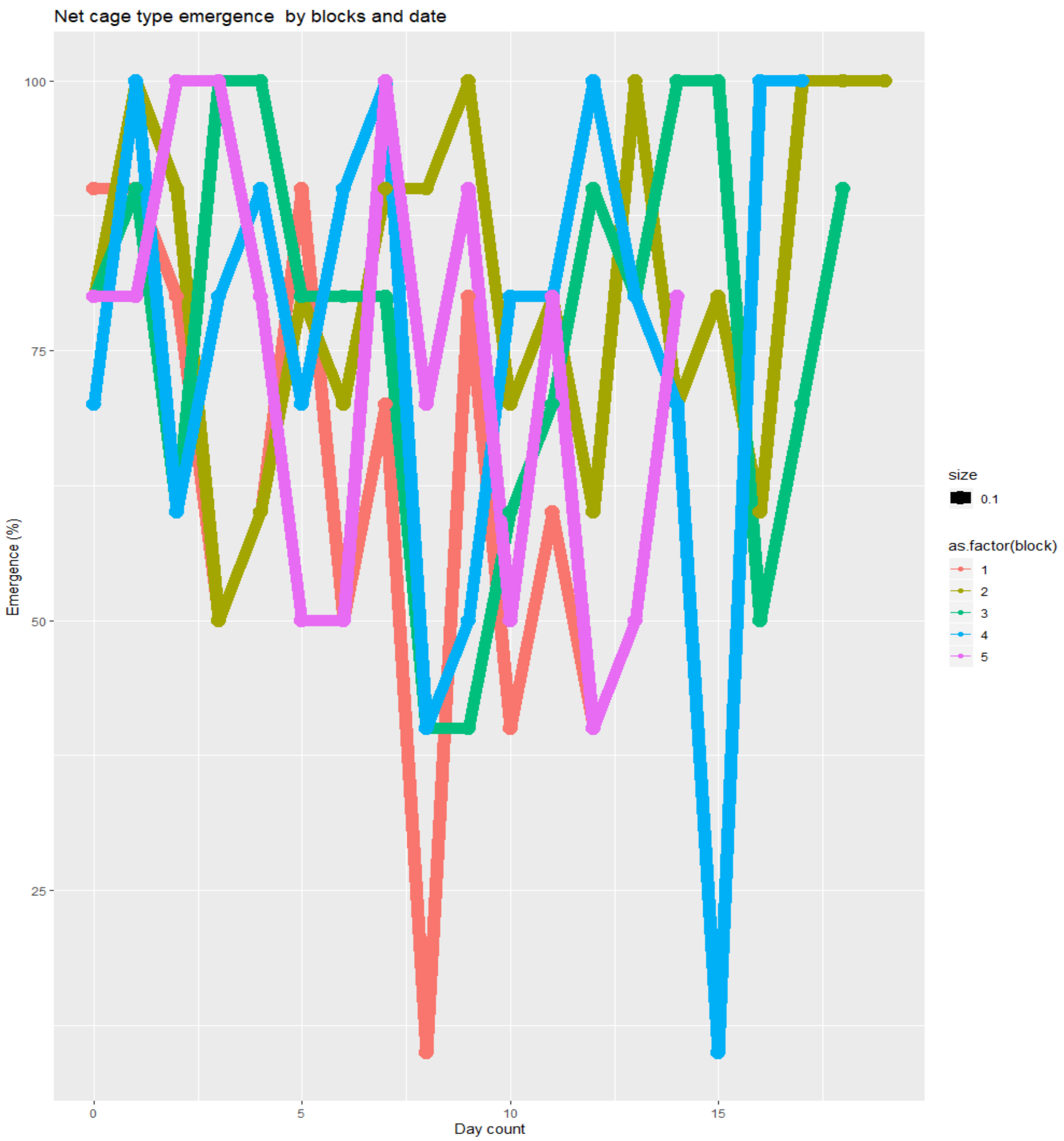


```
> boxplot(mas2$eme2~mas2$tec, data= mas2, col=c("darkcyan", "chocolate1"),
xlab="Cage type",
+         ylab="Emergence (%)")
```

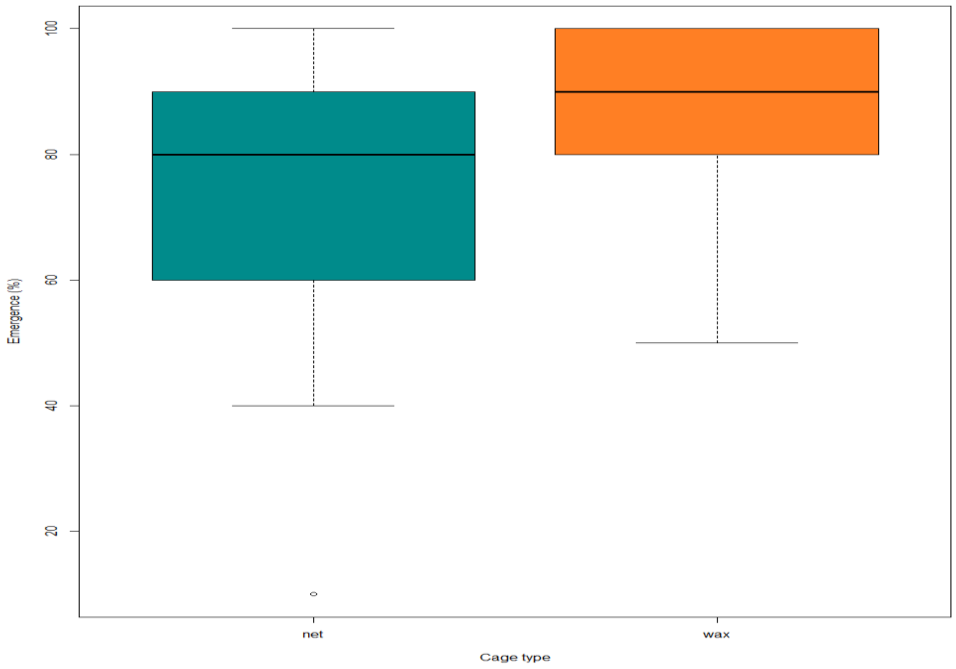


```
Fit model
> model_1 = lme(eme2~ daycount + tec, random = ~1|block,
+                data = mas2_join, method = "ML")
> summary(model_1)
Linear mixed-effects model fit by maximum likelihood
 Data: mas2_join
       AIC     BIC    logLik
  1449.701 1465.35 -719.8503

Random effects:
 Formula: ~1 | block
        (Intercept) Residual
StdDev:    4.348466 16.83005

Fixed effects: eme2 ~ daycount + tec
               Value Std.Error  DF   t-value p-value
(Intercept) 75.55734  3.400499 162 22.219485  0.0000
daycount    -0.13760  0.256092 162 -0.537290  0.5918
tecwax      10.49300  2.613397 162  4.015081  0.0001
 Correlation:
         (Intr) daycnt
daycount -0.609
tecwax   -0.368 -0.022

Standardized Within-Group Residuals:
       Min         Q1        Med         Q3        Max
-3.8578984 -0.5356895  0.2066319  0.6542872  1.5824974

Number of Observations: 169
Number of Groups: 5
```

Best fitted model

```
> model_3 = lme(eme2 ~ tec,
+                    random = ~1|block,
+                    data = mas2_join, method = "ML")
> summary(model_3)
Linear mixed-effects model fit by maximum likelihood
 Data: mas2_join
       AIC      BIC    logLik
  1447.987 1460.507 -719.9935

Random effects:
 Formula: ~1 | block
        (Intercept) Residual
StdDev:    4.177216  16.8585

Fixed effects: eme2 ~ tec
               Value Std.Error  DF   t-value p-value
(Intercept) 74.45847  2.635225 163 28.255068   0e+00
tecwax      10.46381  2.609308 163  4.010184   1e-04
 Correlation:
       (Intr)
tecwax -0.492

Standardized Within-Group Residuals:
       Min         Q1        Med         Q3        Max
-3.9042116 -0.5589838  0.2204854  0.6273616  1.5800396

Number of Observations: 169
Number of Groups: 5
> lsmeans(model_3, pairwise ~ tec) # comparisons of groups
$lsmeans
 tec lsmean   SE df lower.CL upper.CL
 net   74.5 2.64  4     67.1     81.8
 wax   84.9 2.64  4     77.6     92.3

d.f. method: containment
Confidence level used: 0.95

$contrasts
 contrast  estimate   SE  df t.ratio p.value
 net - wax    -10.5 2.61 163 -4.010  0.0001

> comp_ml <- emmeans(model_3, ~tec)
> CLD(comp_ml)
 tec emmean   SE df lower.CL upper.CL .group
 net   74.5 2.64  4     67.1     81.8  1
 wax   84.9 2.64  4     77.6     92.3   2

d.f. method: containment
Confidence level used: 0.95
significance level used: alpha = 0.05
> data_pedicted = mas2_join
> data_pedicted$predictions = predict(model_3, newdata = mas2_join)
> df_means_in_time_pred = aggregate(data_pedicted$predictions, by = list(d
ata_pedicted$daycount,data_pedicted$tec ), FUN = mean)
```

```
> names(df_means_in_time_pred) <- c("day_diff", "tec", "predictions")
> ggplot(data = data_pedicted,
+        aes(x = daycount, y = predictions, color = as.factor(tec), size =
0.1)) +
+   ggtitle("Emergence per cage by blocks and date") +
+   geom_line() +
+   geom_line(aes(daycount,  Emergence, colour=tec), df_means_in_time)
```

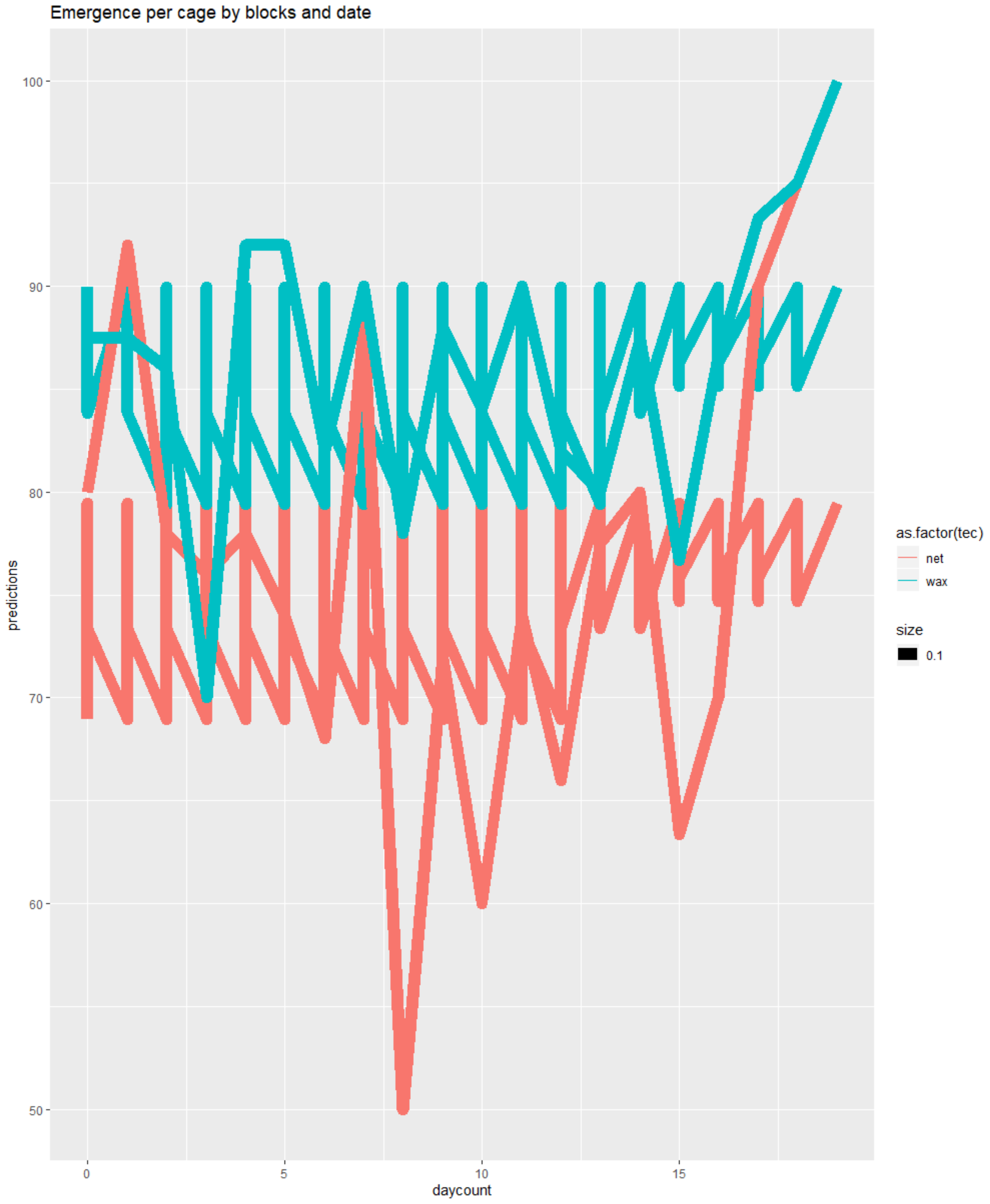

Homoscedasticity

```
> plot(model_3)
```

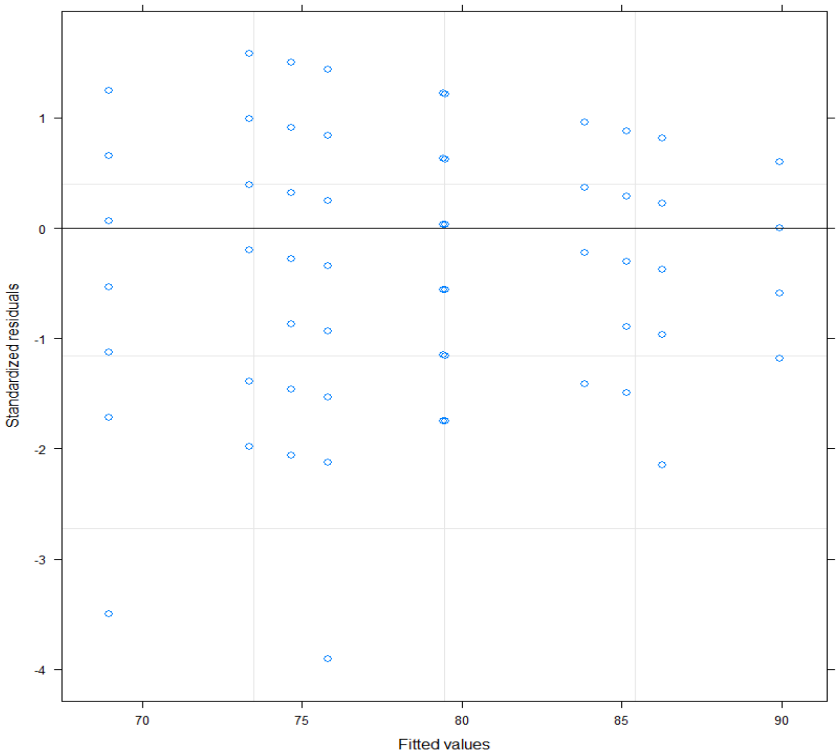


```
> intervals(model_3, which = "fixed")
Approximate 95% confidence intervals

 Fixed effects:
                lower      est.     upper
(Intercept) 69.285769 74.45847 79.63117
tecwax       5.341981 10.46381 15.58563
attr(,"label")
[1] "Fixed effects:"
Normality
> res = model_3$residuals
> ggplot(data = resid(model_3))
> qqnorm(resid(model_3))
> qqline(resid(model_3))
> qqplot(res[,1])
> qqplot(model_3$residuals)
```

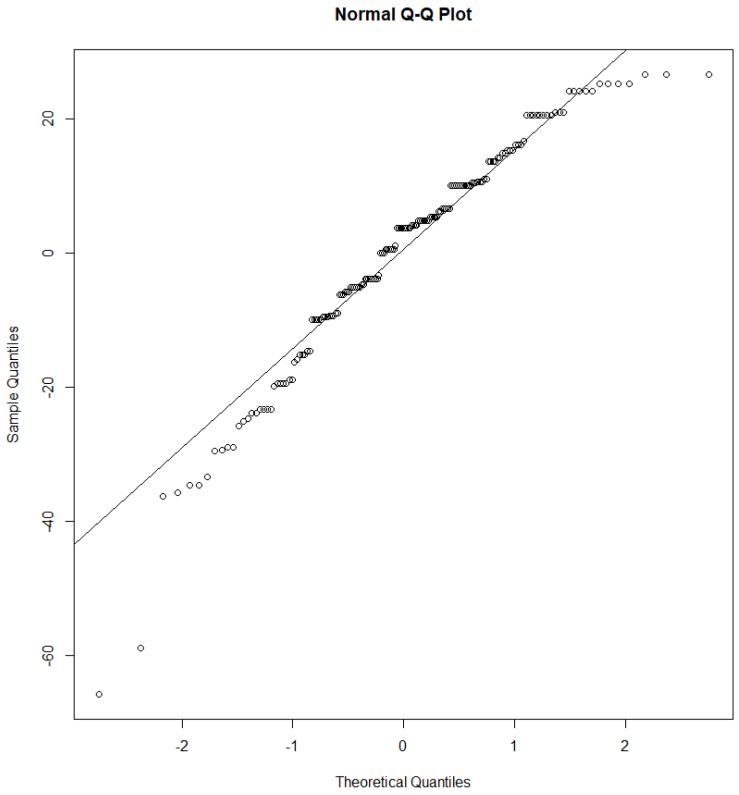


```
> shapiro.test(model_3$residuals)### n
Shapiro-Wilk normality test
data:  model_3$residuals
W = 0.9434, p-value = 4.453e-10

> kruskal.test (eme2 ~ tec, data=mas2_join) #
Kruskal-Wallis chi-squared = 10.221, df = 1, p-value = 0.001388
```

Flyers

```
> group_by(mas2, tec) %>%
+   summarise(
+     count = n(),
+     mean = mean(fly2, na.rm = TRUE),
+     sd = sd(fly2, na.rm = TRUE),
+     median = median(fly2, na.rm = TRUE),
+     IQR = IQR(fly2, na.rm = TRUE))
# A tibble: 2 x 6
  tec   count  mean    sd median   IQR
  <fct> <int> <dbl> <dbl>  <dbl> <dbl>
1 net      85  66.5  24.1     70    40
2 wax      84  77.6  16.5     80    20
```

Descriptive analyses
Wax

```
> mas2_join_wax = mas2[mas2$tec == "wax",]
> ggplot(data = mas2_join_wax,
+        aes(x = daycount, y = fly2 , color = as.factor(block), size =0.1)
) +
+   ggtitle("Wax flyer by blocks and date") +
```

```
+   geom_line()+
+   geom_point()+ xlab("Day count") +
+   ylab("Flyer (%)")
```

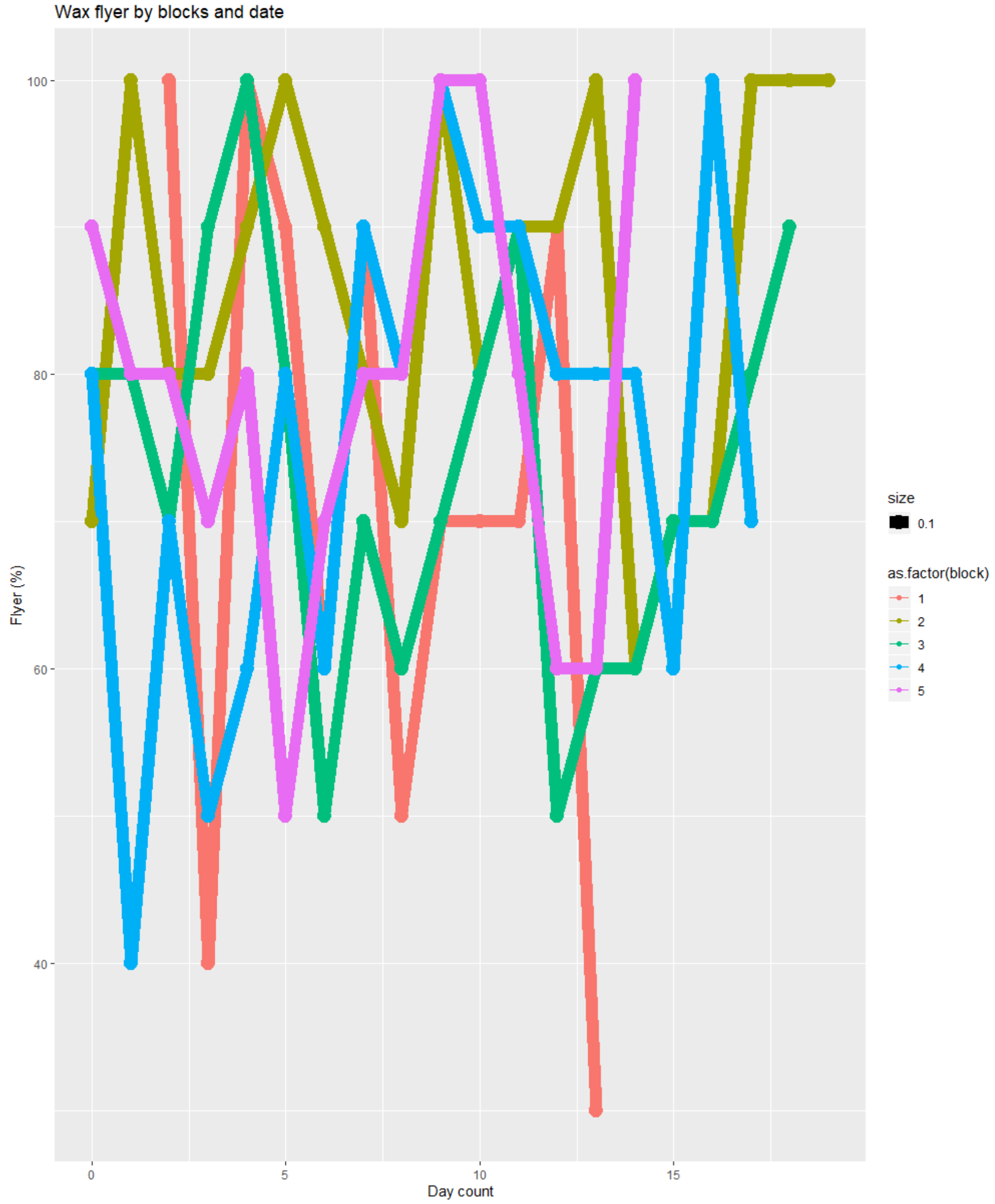

NET

```
> mas2_join_net = mas2[mas2$tec == "net",]
> ggplot(data = mas2_join_net,
+        aes(x = daycount, y = fly2, color = as.factor(block), size =0.1))
+
+   ggtitle("Net flyer by blocks and date") +
+   geom_line()+
+   geom_point()+ xlab("Day count") +
+   ylab("Flyer (%)")
```

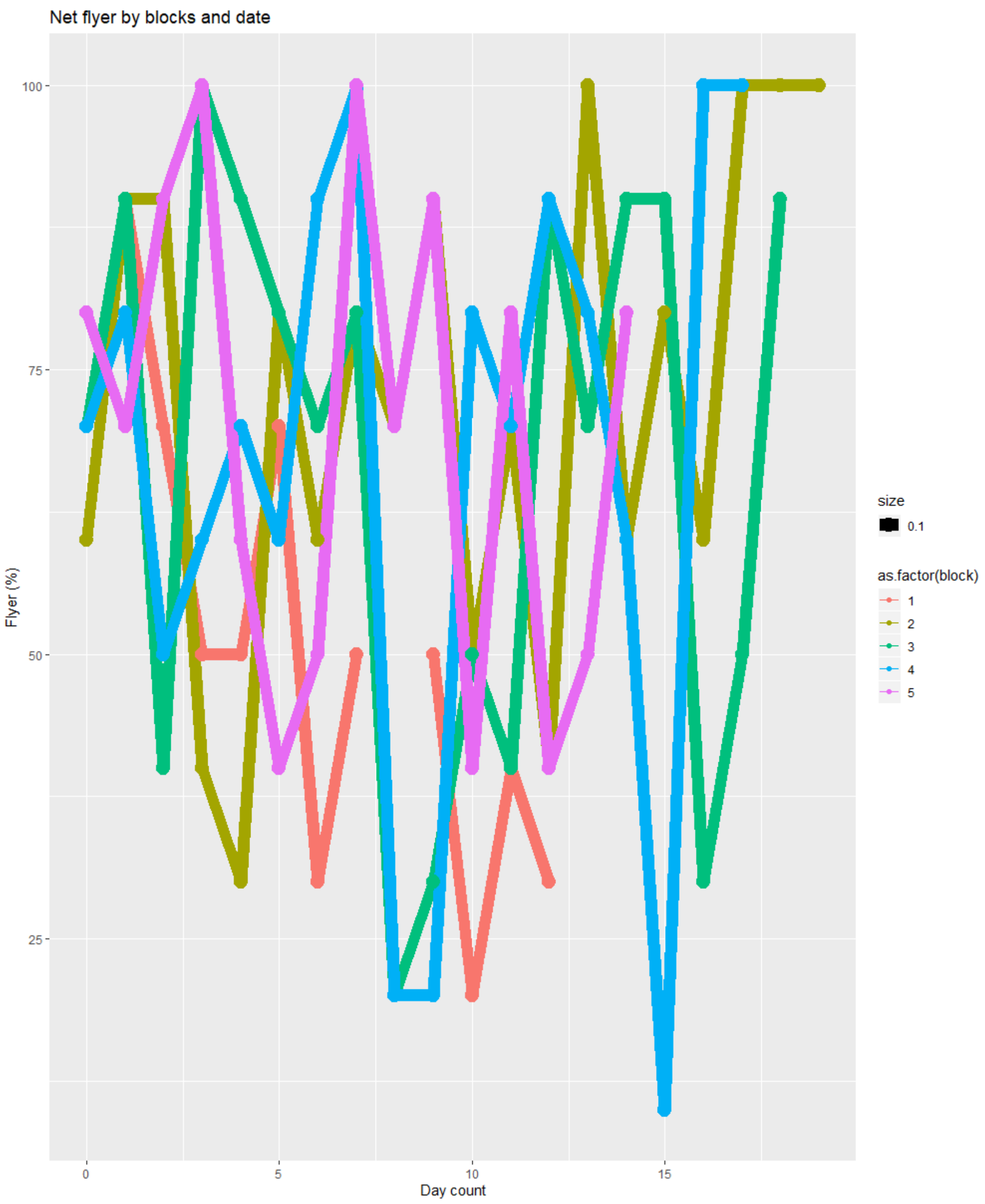


```
> boxplot(mas2$fly2~mas2$tec, data= mas2, col=c("darkcyan", "chocolate1"),
xlab="Cage type",
+         ylab="flyer (%)")
```

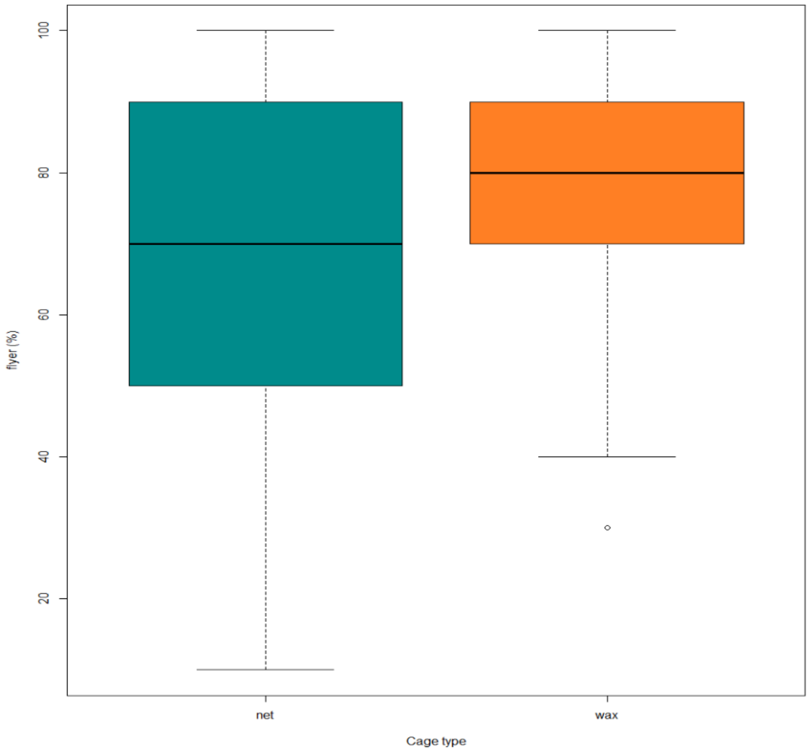


```
Fit model
> model_0 = lme(fly2~ daycount * tec, random = ~1|block,
+               data = mas2_join, method = "ML")
> summary(model_0)
Linear mixed-effects model fit by maximum likelihood
 Data: mas2_join
       AIC      BIC    logLik
  1518.702 1537.481 -753.3509

Random effects:
 Formula: ~1 | block
        (Intercept) Residual
StdDev:    5.084994 20.53746

Fixed effects: fly2 ~ daycount * tec
                   Value Std.Error  DF   t-value p-value
(Intercept)     65.52082  4.797516 161 13.657240  0.0000
daycount        -0.02835  0.436315 161 -0.064972  0.9483
tecwax          10.33028  6.062201 161  1.704047  0.0903
daycount:tecwax  0.17071  0.618580 161  0.275976  0.7829
 Correlation:
                (Intr) daycnt tecwax
daycount        -0.740
tecwax          -0.608  0.583
daycount:tecwax  0.518 -0.696 -0.849

Standardized Within-Group Residuals:
       Min         Q1        Med         Q3        Max
-2.8344174 -0.6732817  0.1045841  0.7465583  1.7148673
```

```
Number of Observations: 169
Number of Groups: 5

Best fitted model
> model_3 = lme(fly2 ~ tec, random = ~1|block, data = mas2_join, method =
"ML")
> summary(model_3)
Linear mixed-effects model fit by maximum likelihood
 Data: mas2_join
       AIC      BIC    logLik
  1514.812 1527.331 -753.4059

Random effects:
 Formula: ~1 | block
        (Intercept) Residual
StdDev:    5.108367 20.54237

Fixed effects: fly2 ~ tec
               Value Std.Error  DF   t-value p-value
(Intercept) 65.28273  3.216979 163 20.293179   0e+00
tecwax      11.76381  3.179488 163  3.699909   3e-04
 Correlation:
       (Intr)
tecwax -0.491

Standardized Within-Group Residuals:
       Min         Q1        Med         Q3        Max
-2.8312404 -0.6233080  0.1362410  0.7472186  1.7208162

Number of Observations: 169
Number of Groups: 5
> lsmeans(model_3, pairwise ~ tec) # comparisons of groups
$lsmeans
 tec lsmean   SE df lower.CL upper.CL
 net   65.3 3.22  4     56.4     74.2
 wax   77.0 3.23  4     68.1     86.0

d.f. method: containment
Confidence level used: 0.95

$contrasts
 contrast  estimate   SE  df t.ratio p.value
 net - wax    -11.8 3.18 163 -3.700  0.0003

> comp_ml <- emmeans(model_3, ~tec)
> CLD(comp_ml)
 tec emmean   SE df lower.CL upper.CL .group
 net   65.3 3.22  4     56.4     74.2  1
 wax   77.0 3.23  4     68.1     86.0   2

d.f. method: containment
Confidence level used: 0.95
significance level used: alpha = 0.05
> data_pedicted = mas2_join
```

```
> data_pedicted$predictions = predict(model_3, newdata = mas2_join)
> df_means_in_time_pred = aggregate(data_pedicted$predictions, by = list(d
ata_pedicted$daycount,data_pedicted$tec ), FUN = mean)
> names(df_means_in_time_pred) <- c("day_diff", "tec", "predictions")
> ggplot(data = data_pedicted,
+        aes(x = daycount, y = predictions, color = as.factor(tec), size =
0.1)) +
+   ggtitle("Flyer per cage by blocks and date") +
+   geom_line() +
+   geom_line(aes(daycount,  flyer, colour=tec), df_means_in_time)
```

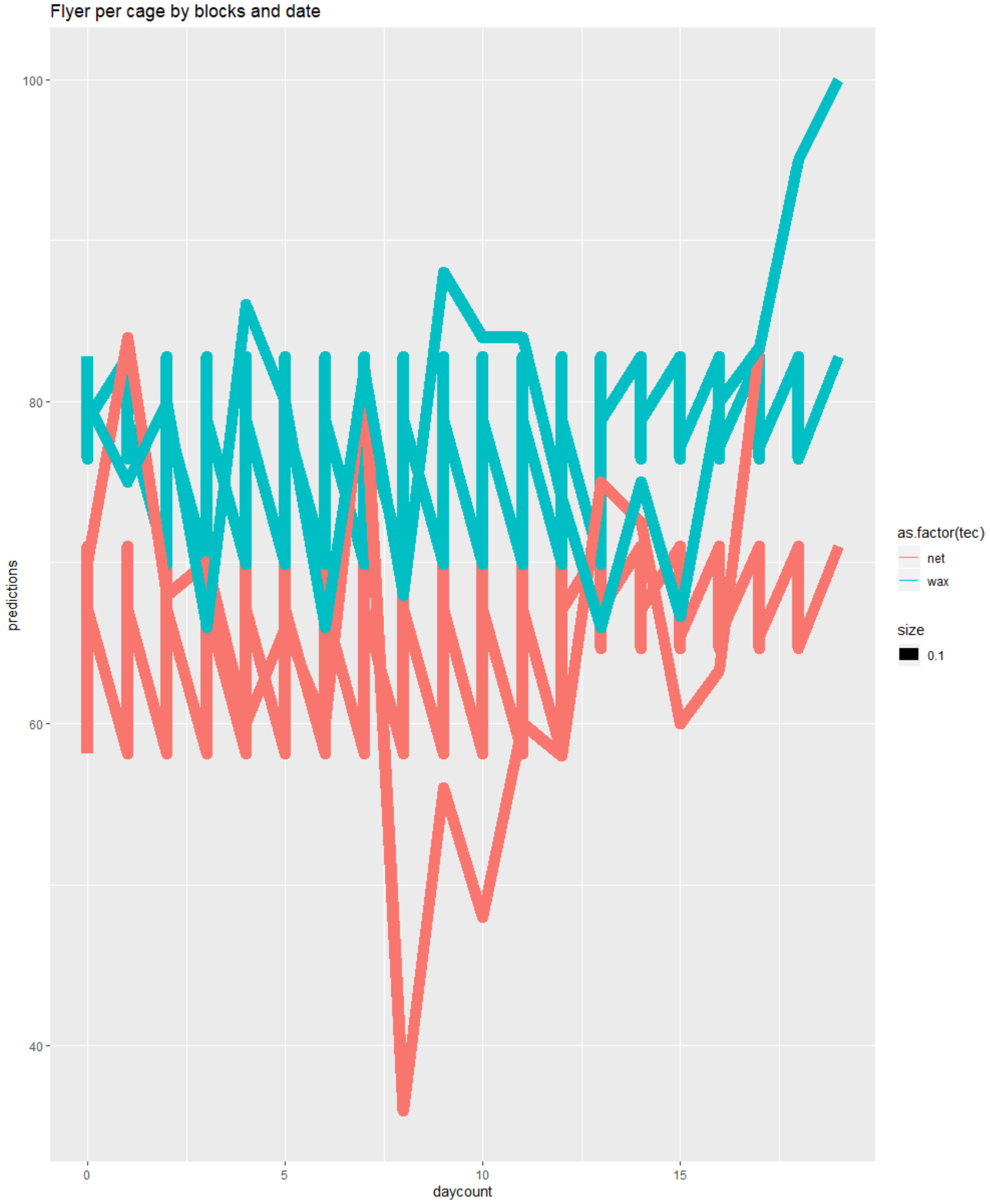

Homoscedasticity

```
> plot(model_3)
```

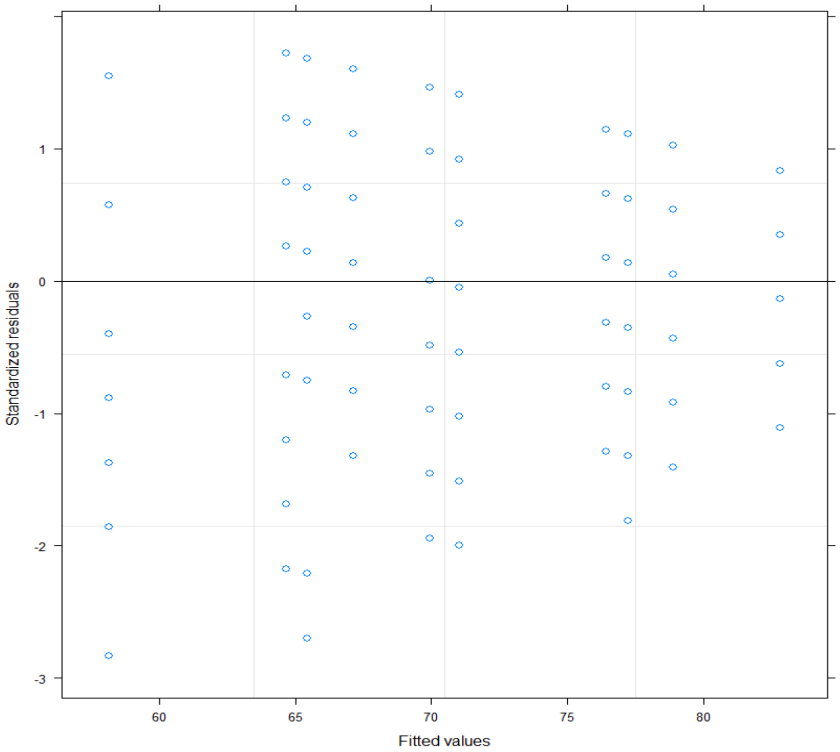


```
> intervals(model_3, which = "fixed")
Approximate 95% confidence intervals

 Fixed effects:
                lower     est.    upper
(Intercept) 58.96811 65.28273 71.59736
tecwax       5.52278 11.76381 18.00485
attr(,"label")
[1] "Fixed effects:"

Normality
> res = model_3$residuals
> ggplot(data = resid(model_3))
> hist(, breaks =  20)
> qqnorm(resid(model_3))
> qqline(resid(model_3))
> qqplot(res[,1])
> qqplot(model_3$residuals)
```

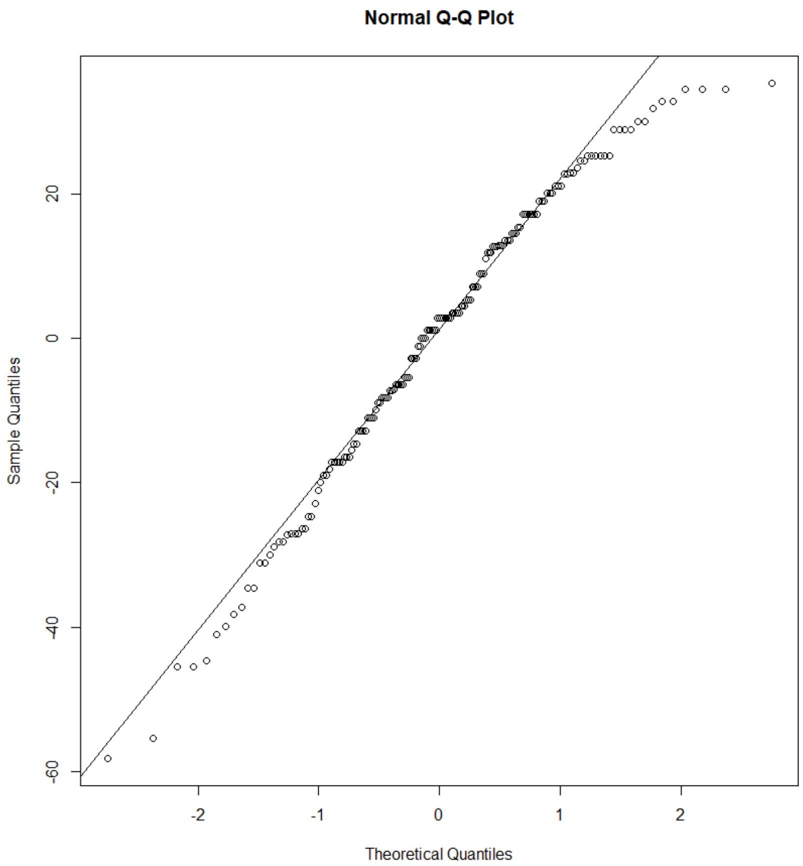


```
> shapiro.test(model_3$residuals)
Shapiro-Wilk normality test
data:  model_3$residuals
W = 0.97159, p-value = 3.393e-06
> kruskal.test (fly2 ~ tec, data=mas2_join)
Kruskal-Wallis chi-squared = 8.7786, df = 1, p-value = 0.003048
```

S2 Dataset all parameters.

System	net	net	net	net	net	wax	wax	wax	wax	wax
Replicas	Egg production [ml]	Number of egg hatch	Larva develop [days]	Number of pupae recovery	Number of adult emergence	Egg production [ml]	Number of egg hatch	Larva develop [days]	Number of pupae recovery	Number of adult emergence
1	0.2	72	7	66	63	0.05	16	8	10	10
1	0.2	44	7	32	31	0.1	84	7	60	54
1	0.4	34	7	23	21	0.25	84	7	60	55
1	0.8	30	7	12	13	0.4	65	7	41	40
1	0.4	21	7	7	6	0.4	79	7	58	53
1	0.8	31	7	25	24	0.5	76	7	55	43
1	0.5	56	6	39	38	0.7	75	7	52	42
1	0.7	14	7	5	5	0.4	77	7	64	50
1	0.7	32	7	23	21	0.9	82	6	63	49
1	0.6	50	7	24	22	0.8	78	7	56	41
1	0.7	45	9	17	18	0.9	72	9	52	54
1	0.3	37	8	13	12	0.6	70	8	49	44
1	0.3	26	7	8	7	0.4	77	7	63	57
1	0.2	31	7	22	22	0.4	70	7	49	43
1	0.3	10	NA	1	1	0.3	58	8	27	22
1	0.1	17	NA	3	3	0.4	68	NA	36	33
1	0.1	32	NA	10	8	0.3	50	NA	25	32
2	0.2	86	NA	64	59	0.09	66	NA	46	39
2	0.5	4	NA	4	4	0.2	53	NA	41	41
2	0.5	12	6	5	4	0.2	60	6	41	35
2	0.6	21	6	7	2	0.3	57	6	31	25
2	0.6	10	8	3	3	0.4	52	7	40	36
2	0.9	17	8	13	12	0.6	64	7	48	47
2	0.7	10	NA	6	5	1	78	8	66	66
2	0.6	12	7	7	7	0.9	63	7	41	38
2	0.6	38	7	34	31	1	56	7	27	26
2	0.4	94	7	61	59	1	31	7	18	16
2	0.6	12	7	1	1	0.5	44	7	39	32
2	0.4	20	8	9	8	0.75	41	8	15	15
2	0.4	25	8	13	13	0.9	25	8	19	19
2	0.3	67	7	46	37	0.5	46	7	31	23
2	0.5	52	8	18	13	0.5	33	8	11	11
2	0.25	62	7	42	39	0.55	30	7	19	19
2	0.2	65	7	43	41	0.6	52	7	33	15
2	0.35	16	NA	1	1	0.55	43	8	22	22
2	0.2	20	8	8	9	0.5	29	8	8	7
2	0.1	50	8	21	20	0.2	45	8	30	19
3	0.1	64	8	41	39	0.05	66	8	49	46
3	0.3	62	8	49	48	0.1	48	8	19	18
3	0.6	22	7	14	11	0.35	28	7	13	11

3	0.4	52	7	29	23	0.6	36	7	19	17
3	0.6	24	7	5	4	0.7	45	7	3	3
3	0.6	26	7	12	12	0.7	75	8	43	39
3	0.4	80	7	68	63	0.6	39	9	21	19
3	0.7	30	8	9	7	0.6	18	8	14	14
3	0.2	55	7	39	33	0.4	70	8	56	53
3	0.5	35	7	26	24	0.8	49	8	30	29
3	0.7	14	7	3	3	0.75	75	8	57	51
3	0.5	29	7	17	13	0.8	67	8	52	44
3	0.5	70	7	51	46	0.5	74	8	63	60
3	1.2	29	7	5	5	0.6	49	7	39	37
3	0.4	43	7	27	26	0.4	37	7	30	28
3	0.4	69	6	56	37	0.3	58	6	37	33
3	0.3	79	6	58	56	0.4	45	8	33	31
3	0.3	30	6	5	3	0.1	57	6	38	30
3	0.2	42	8	22	22	0.2	38	8	24	21
4	0.01	47	8	28	26	0.01	66	8	35	41
4	0.15	25	8	20	18	0.15	94	8	91	57
4	0.2	28	9	14	13	0.2	81	7	68	62
4	0.2	52	8	36	31	0.3	74	7	61	55
4	0.4	14	10	5	5	0.4	51	9	28	30
4	0.6	24	9	14	12	0.6	52	8	25	31
4	0.5	34	7	24	20	0.65	67	9	39	42
4	0.3	70	7	44	36	0.6	32	10	9	8
4	0.4	50	7	28	24	0.7	59	8	23	22
4	0.6	50	7	33	NA	0.7	23	NA	12	12
4	0.3	82	6	68	63	0.6	58	6	30	26
4	0.7	37	8	20	18	0.7	46	8	16	17
4	0.5	80	7	74	68	0.7	43	9	17	10
4	0.5	82	7	64	54	0.7	56	7	42	30
4	0.5	90	7	71	67	0.6	73	NA	37	40
4	0.6	51	9	28	25	0.5	57	8	34	32
4	0.55	52	8	30	31	0.6	51	8	34	31
4	0.6	66	10	12	9	0.5	67	10	45	36
4	0.6	54	9	18	9	0.6	46	9	21	18
4	0.3	45	8	33	30	0.4	46	8	18	14
4	0.2	79	7	51	42	0.3	52	8	16	15
4	0.2	67	7	33	30	0.3	48	7	21	15
5	0.05	69	9	49	43	0.05	78	9	66	75
5	0.25	35	9	28	26	0.2	25	9	25	22
5	0.4	40	8	16	16	0.4	59	8	35	24
5	0.5	55	8	47	55	0.6	62	7	48	47
5	0.5	36	8	18	16	0.5	73	8	40	38
5	0.4	31	8	13	9	0.6	61	8	39	36
5	0.3	34	8	25	20	0.6	62	8	47	24
5	0.5	39	8	27	22	0.6	70	7	50	46

5	0.6	45	8	25	24	0.8	75	8	52	48
5	0.6	23	NA	9	9	0.8	71	NA	44	41
5	0.7	16	NA	5	5	0.6	56	NA	32	30
5	0.7	17	NA	9	8	0.4	53	NA	43	40
5	0.5	21	NA	16	15	0.5	64	NA	50	42
5	0.3	63	NA	48	42	0.4	67	NA	59	57
5	0.3	70	NA	38	38	0.3	65	NA	21	26
5	0.2	80	NA	75	61	0.2	80	NA	70	48
Grand Total	40.86	4058	605	2525	2246	46.4	5386	618	3529	3145

S3. Dataset quality control parameters.

System		net	net	wax	wax
Replicas	Number of pupae	Number of emerged	Number of flyer	Number of emerged	Number of flyer
1	10	9	7	10	10
1	10	9	9	5	4
1	10	8	7	10	10
1	10	5	5	9	9
1	10	6	5	NA	NA
1	10	9	7	8	6
1	10	5	3	9	9
1	10	7	5	6	5
1	10	1	0	7	7
1	10	8	5	7	7
1	10	4	2	8	7
1	10	6	4	9	9
1	10	4	3	7	3
2	10	8	6	8	7
2	10	10	9	10	10
2	10	9	9	10	8
2	10	5	4	8	8
2	10	6	3	9	9
2	10	8	8	10	10
2	10	7	6	9	9
2	10	9	8	9	8
2	10	9	7	10	7
2	10	10	9	10	10
2	10	7	5	8	8
2	10	8	7	10	9
2	10	6	4	10	9
2	10	10	10	10	10
2	10	7	6	8	6
2	10	8	8	7	7
2	10	6	6	8	7
2	10	10	10	10	10
2	10	10	10	10	10

2	10	10	10	10	10
3	10	8	7	9	8
3	10	9	9	9	8
3	10	6	4	7	7
3	10	10	10	9	9
3	10	10	9	10	10
3	10	8	8	9	8
3	10	8	7	7	5
3	10	8	8	9	7
3	10	4	2	6	6
3	10	4	3	7	7
3	10	6	5	8	8
3	10	7	4	10	9
3	10	9	9	8	5
3	10	8	7	8	6
3	10	10	9	8	6
3	10	10	9	9	7
3	10	5	3	8	7
3	10	7	5	8	8
3	10	9	9	9	9
4	10	7	7	9	8
4	10	10	8	8	4
4	10	6	5	8	7
4	10	8	6	5	5
4	10	9	7	9	6
4	10	7	6	10	8
4	10	9	9	9	6
4	10	10	10	9	9
4	10	4	2	9	8
4	10	5	2	10	10
4	10	8	8	9	9
4	10	8	7	9	9
4	10	10	9	8	8
4	10	8	8	9	8
4	10	7	6	9	8
4	10	1	1	7	6
4	10	10	10	10	10
4	10	10	10	10	7
5	10	8	8	9	9
5	10	8	7	8	8
5	10	10	9	8	8
5	10	10	10	8	7
5	10	8	6	8	8
5	10	5	4	8	5
5	10	5	5	8	7
5	10	10	10	9	8

5	10	7	7	8	8
5	10	9	9	10	10
5	10	5	4	10	10
5	10	8	8	8	8
5	10	4	4	6	6
5	10	5	5	6	6
5	10	8	8	10	10
Grand Total	860	637	559	718	652
